# Nanoimprint Lithography Enabling High-Performance Organic Optoelectronics: Advances and Perspectives

**DOI:** 10.1007/s40820-026-02093-z

**Published:** 2026-02-04

**Authors:** Ningning Song, Xinghao Guo, Hongqiao Zhao, Bohang Li, Ningning Liang, Tianrui Zhai

**Affiliations:** School of Physics and Optoelectronic Engineering, Beijing University of Technology, Beijing, 100124 People’s Republic of China

**Keywords:** Nanoimprint lithography, Organic optoelectronics, Light management, Flexible electronics, Micro/nanostructuring

## Abstract

Organic optoelectronic devices demonstrate immense potential in flexible displays, wearable electronics, and artificial skin, needing precise light-field and morphology management strategies to further improve their opto-electric performance. Nanoimprint lithography (NIL) has emerged as a high-resolution, high-efficiency, and low-cost patterning technique that mechanically transferring micro/nanoscale patterns from a template to a substrate to significantly enhance the optoelectronic performance through the precise creation of advanced light-management structures, combined with additional solid-state stacking morphology. This review systematically summarizes recent advances in NIL technology for organic optoelectronics. It begins with an introduction to the fundamental principles, main process variants (thermal, ultraviolet, and electrochemical NIL), as well as key technical issues. Subsequently, through specific applications in organic light-emitting diodes, organic solar cells, and organic field-effect transistors, it highlights the exceptional capabilities of NIL to enhance device performance by controlling crystallization and creating functional micro/nanostructuring. Specific advantages include enabling high-efficiency light management to overcome efficiency bottlenecks, facilitating low-cost, high-throughput manufacturing for industrialization, full compatibility with flexible substrates for emerging applications, enabling multifunctional integration and novel device architectures, and tailoring material microstructures and properties advance fundamental research. Finally, we discuss the remaining challenges and future prospects of NIL in integrated organic optoelectronic systems.

## Highlights

- Nanoimprint lithography (NIL) enables high-performance light management in organic light-emitting diodes and organic solar cells, and enhances charge transport in organic field-effect transistors via controlled molecular ordering, pushing organic optoelectronics beyond conventional efficiency limits.
- The technology provides a scalable, low-cost platform for large-area fabrication on flexible substrates, effectively bridging the gap between laboratory innovation and industrial mass production.
- NIL uniquely empowers the creation of multifunctional integrated devices and novel architectures, opening pathways for next-generation wearable electronics and bio-integrated systems.

## Graphical abstract

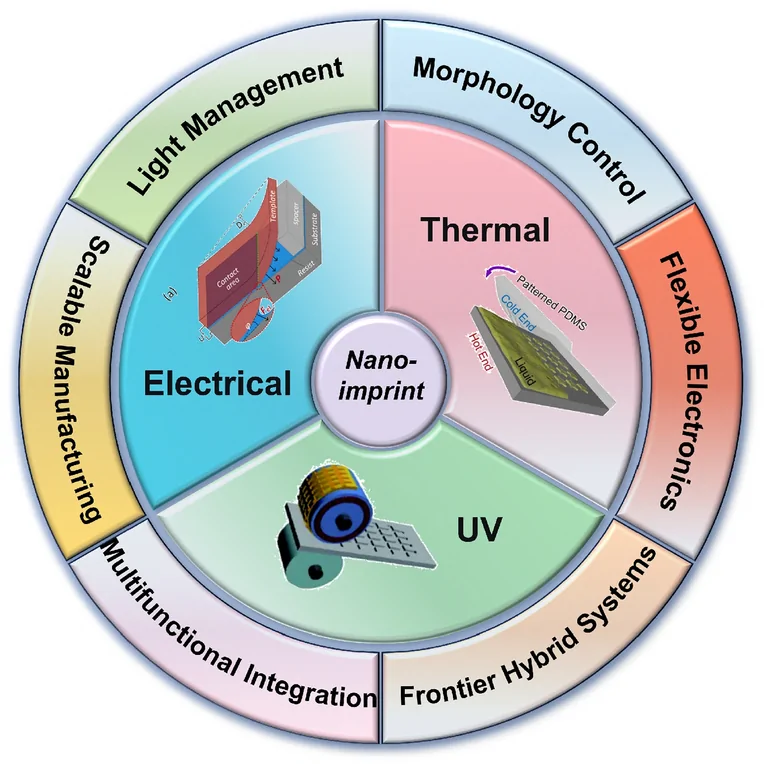

## Introduction

Organic semiconductors have opened new frontiers in optoelectronics due to their exceptional mechanical flexibility, low-temperature processability, large-area manufacturability, lightweight nature, and low cost. These advantages make organic light-emitting diodes (OLEDs) [1–7], organic solar cells (OPVs) [8–15], and organic field-effect transistors (OFETs) [16–23] highly promising technologies. Organic materials are abundant, inexpensive, compatible with roll-to-roll (R2R) processing, and intrinsically flexible [24–26]. Significant improvements in device efficiency and operational stability in recent years [27, 28] have accelerated their commercialization in displays, optical communications, biomedicine, building-integrated photovoltaics, agricultural greenhouses, consumer electronics, as well as e-paper, sensors, and memory devices [29–34]. Nevertheless, achieving high-performance devices that simultaneously satisfy requirements for large-area coverage, high resolution, mechanical flexibility, and low cost remains challenging. Specific limitations persist: OLEDs face low light extraction efficiency [35–37], OPVs exhibit insufficient light absorption and charge transport capabilities [38, 39], while OFETs are constrained by limited carrier mobility and device uniformity [40, 41]. Addressing these issues demands advanced micro/nanofabrication technologies that combine high precision, scalability, and broad material compatibility.

Micro/nano-optical structures obtained by the methods of photolithography, electron beam lithography, focused ion beam etching, nanoimprint lithography and scanning probe lithography, play a pivotal role in organic optoelectronic devices through mechanisms such as light scattering, localized field enhancement, and light trapping [9, 42]. These structures enable enhanced light absorption in organic photovoltaics, improved light outcoupling efficiency in organic light-emitting diodes, and optimized charge mobility in organic electric field transistor. However, practical applications face multiple challenges, including requirements for scalability and uniformity in large-area fabrication, process compatibility with flexible substrates and multilayer device architectures, balancing optical and electrical performance, cost-effectiveness considerations, and long-term structural stability. Achieving scalable implementation thus requires further development of scalable nanofabrication and integration technologies, along with multidisciplinary co-design to optimize opto-electro-structural performance [1, 9]. Nanoimprint lithography (NIL) provides critical technical support for organic optoelectronic devices through its high-precision manufacturing capability that breaks the optical diffraction limit, large-area production compatibility with R2R processes, and excellent material adaptability [43, 44]. This technology enables simultaneous multidimensional functionality including optical modulation, electrical optimization, and interface morphology construction, while significantly reducing costs through reusable molds and simplified processes. Such a “structure-performance” integrated manufacturing strategy effectively resolves the fundamental conflict between device efficiency and scalable fabrication, offering a viable pathway for the industrial development of organic optoelectronics [18, 19].

It is first introduced by Chou et al. in 1995 [45], has emerged as a key enabling technology in this regard. Its core principle is analogous to traditional “stamping” or “printing,” replicating micro/nanostructures from a mold onto a target material through mechanical deformation. It relies on mechanical deformation to replicate micro/nanostructures with high resolution, high throughput, and low cost, making it especially suitable for solvent-sensitive, thermally sensitive, and optically functional organic materials. Unlike laser direct writing (low speed and depth-of-focus issues) [46], scanning probe lithography (low throughput) [47], interference lithography (restricted to periodic patterns) [48], NIL bypasses the optical diffraction limit and the constraints of scanning, providing exceptional process flexibility and scalability. It should be noted that directed self-assembly strategies, particularly those employing colloidal nanoparticles within physical or chemical templates, have also emerged as powerful routes for generating periodic and complex nanostructures with high yield and precision [49]. These bottom-up approaches share several advantages with NIL, including scalability and compatibility with solution-processable organic materials. However, NIL offers distinct advantages in deterministic patterning of arbitrary, non-periodic geometries over large areas, which is often crucial for specific light-management and device architectures in organic optoelectronics. It is compatible with various organic polymers, liquid inks, nanomaterials, and composites [50–54]. Significantly, this compatibility also extends to emerging organic–inorganic hybrid materials (e.g., perovskite precursors and sol–gel matrices), whose solution processability and low-temperature curing are well-suited to NIL techniques. This versatility enables NIL to meet the requirements for producing complex micro/nanostructures not only in conventional OLEDs, OPVs, and OFETs but also in next-generation hybrid optoelectronic devices.

**Fig. 1:**
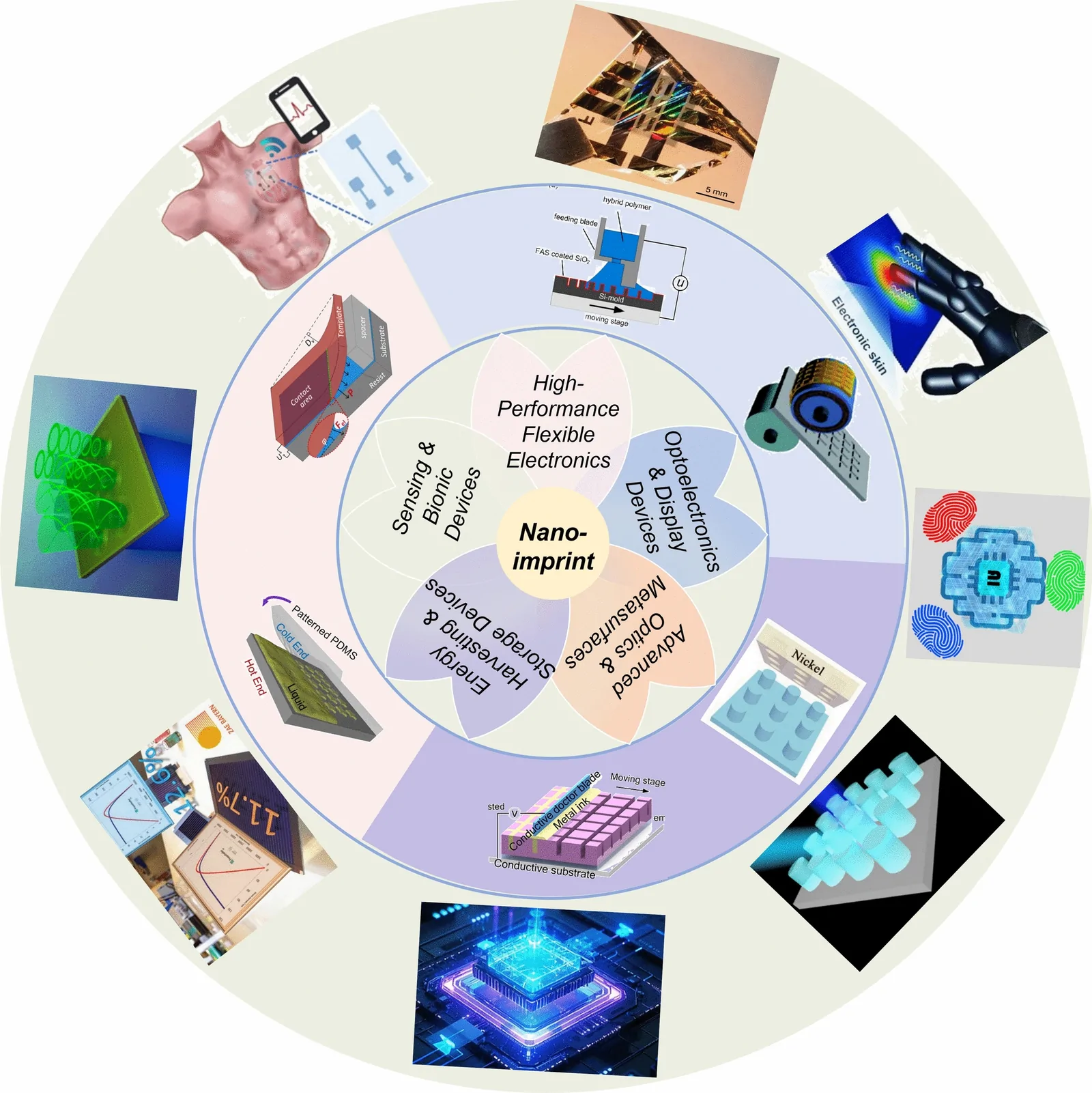
Applications of nanoimprint technology across various fields highlight its versatility and impact in different domains [9, 55–59]. Reproduced with permission. [9] Copyright 2018, Springer Nature. Reproduced with permission. [55] Copyright 2019, Wiley. Reproduced with permission. [56] Copyright 2025, Springer Nature. Reproduced with permission. [57] Copyright 2010, Wiley. Reproduced with permission. [58] Copyright 2008, Wiley. Reproduced with permission. [59] Copyright 2013, Springer Nature

This review comprehensively outlines the development of NIL technology, detailing its operational principles, recent advances, and persistent challenges. It highlights representative achievements in NIL-fabricated OLEDs, OPVs, and OFETs, emphasizing its pivotal role in enabling high-efficiency light management to break through efficiency bottlenecks, facilitating low-cost and high-throughput manufacturing to accelerate industrialization, ensuring full compatibility with flexible substrates for pioneering applications in wearable and stretchable electronics, empowering multifunctional integration and novel device architectures, and precisely controlling material microstructure to advance fundamental research. As demand for photonic and optoelectronic devices continues to grow, NIL has demonstrated substantial progress over the past two decades across disciplines including optics, materials science, nanofabrication, optoelectronics, and electronics (Fig. 1) [9, 55–59], showing great potential to drive innovation in next-generation organic devices.

**Fig. 2:**
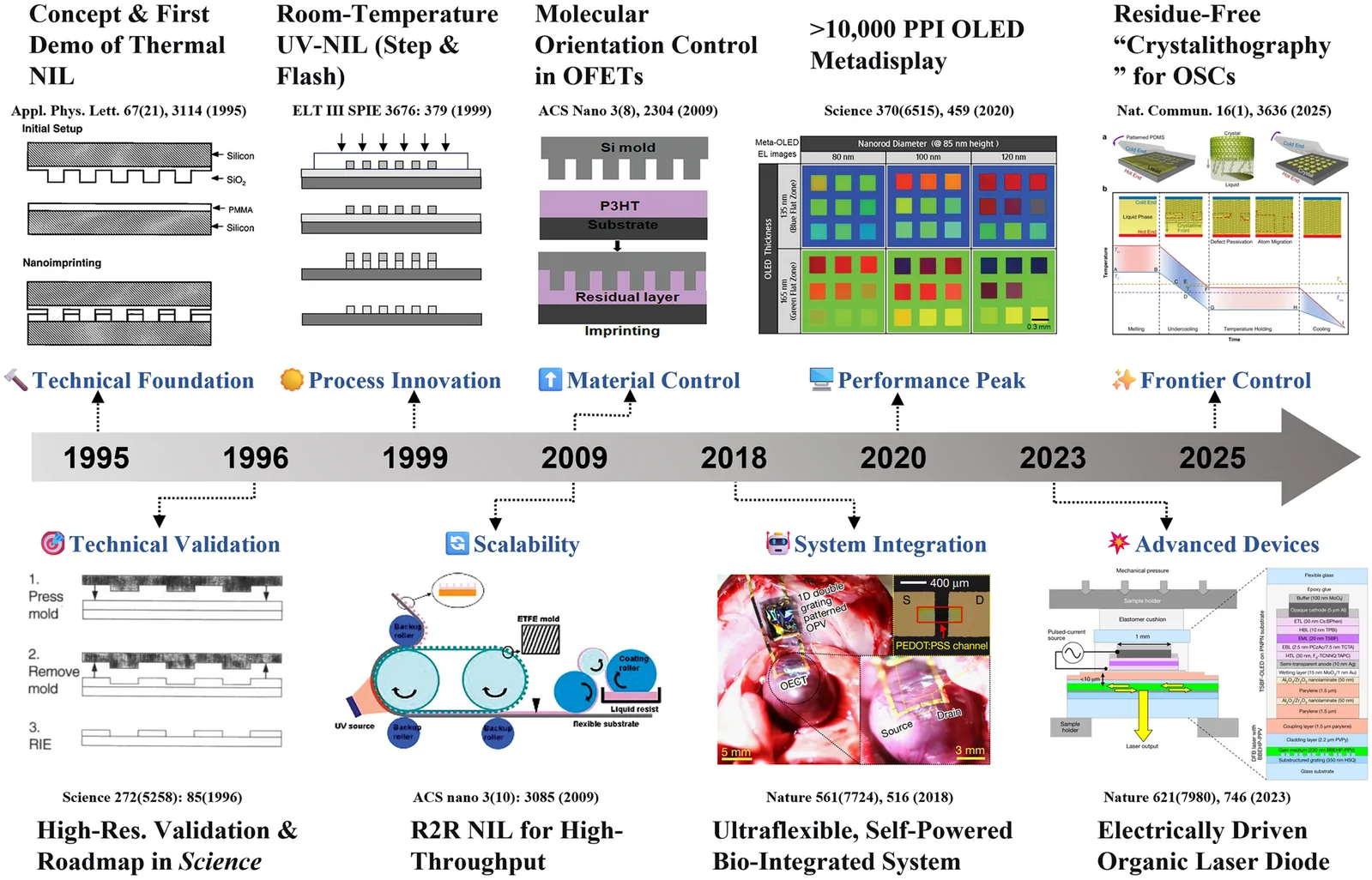
Key milestones in the development of nanoimprint lithography (NIL). The timeline highlights pivotal advances, from the initial demonstration and process innovations (e.g., UV-NIL, R2R NIL) to breakthroughs in material control and, ultimately, the realization of complex integrated systems such as ultra-high-resolution displays and electrically driven lasers. Reproduced with permission. [45] Copyright 1995, AIP Publishing. Reproduced with permission. [60] Copyright 1996, American Association for the Advancement of Science. Reproduced with permission. [61] Copyright 1999, SPIE. Reproduced with permission. [62] Copyright 2009, American Chemical Society. Reproduced with permission. [63] Copyright 2009, American Chemical Society. Reproduced with permission. [9] Copyright 2018, Springer Nature. Reproduced with permission. [64] Copyright 2020, American Association for the Advancement of Science. Reproduced with permission. [65] Copyright 2023, Springer Nature. Reproduced with permission. [56] Copyright 2025, Springer Nature

To encapsulate this evolution and provide a structured perspective on its technological journey, Fig. 2 charts the key milestones in NIL development over the past three decades [9, 45, 56, 60–65]. This timeline not only chronicles the transition from foundational demonstrations (e.g., thermal NIL) to process innovations that enabled scalability and gentler processing (e.g., UV and R2R NIL) but also highlights pivotal moments where NIL’s unique capabilities—such as controlling molecular order and enabling novel device architectures—catalyzed breakthroughs in organic optoelectronics, from ultra-high-resolution displays to self-powered bio-integrated systems. Having established this historical and developmental context, we now delve into the fundamental principles and classical techniques that underpin NIL’s versatility. The subsequent sections will dissect the physical processes, material considerations, and specific variants of NIL, before exploring its transformative applications in OLEDs, OPVs, and OFETs.

## Classical Technologies and Physical Process in NIL Technology

Micro/nanopatterning technologies have been widely used for constructing micro/nanostructures in organic optoelectronic devices. Photolithography is the cornerstone of modern integrated circuit manufacturing, capable of achieving incredibly high resolution (feature sizes below 10 nm) and, in the context of display production, of patterning over very large, meter-scale substrates [53, 66]. However, its direct application to the fabrication of organic optoelectronic devices faces significant challenges due to incompatibility with processing steps. The high-temperature baking steps, aggressive chemical developers and strippers, and often high-energy radiation inherent in standard photolithographic processes can degrade sensitive organic semiconductors, damage flexible plastic substrates, and compromise the integrity of multilayer device architectures. Furthermore, the high capital and operational costs of advanced lithography tools are difficult to justify for many cost-sensitive, large-area organic electronic applications. Consequently, while indispensable in silicon chip fabs, these techniques are primarily used as research tools in the development of organic optoelectronics rather than as viable routes for low-cost, flexible mass manufacturing.

In contrast, NIL is a nanopatterning technology characterized by high efficiency, high resolution, and large-area applicability [44]. Its core principle relies on mechanical deformation to replicate micro- and nanoscale structures from a mold onto a substrate, bypassing the optical diffraction limit and the serial writing constraints of techniques such as photolithography or electron-beam lithography.

### Physical Process and Basic Mechanism

**Fig. 3:**
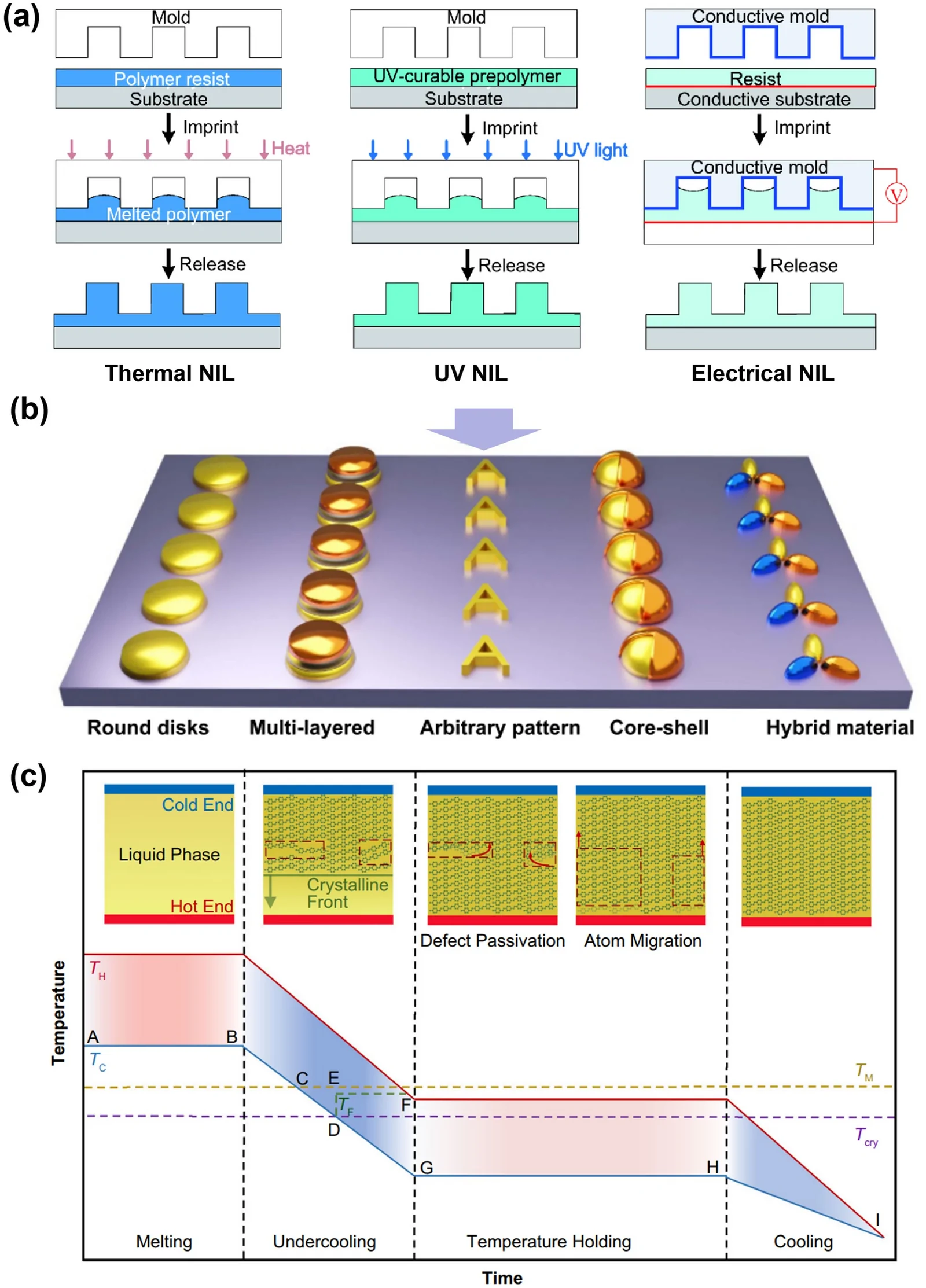
Flowchart of nanoimprint lithography techniques: **a** Schematic diagrams of thermal NIL, UV NIL, and electro-NIL technologies. Reproduced with permission. [53] Copyright 2019, Springer Nature. **b** Diverse shapes and compositions of units. Reproduced with permission. [68] Copyright 2024, IOP Publishing. **c** Schematic illustration of material transformation mechanisms during imprinting, with internal diagrams showing the evolution of molecular structures at different stages. Reproduced with permission. [56] Copyright 2025, Springer Nature

The fundamental procedure of NIL consists of a three-step cycle: imprinting, curing (or solidification), and demolding. Herein, the mechanistic process of preparing organic semiconductor crystals via NIL technology is emphatically introduced as illustrated in Fig. 3c [56]. The organic powder is first loaded onto a target substrate, heated to a liquid phase by controlling the substrate temperature, and then a patterned polydimethylsiloxane (PDMS) mold is applied to the organic material. Under external pressure and capillary force, the molecules are shaped accordingly. Finally, the mold is cooled, and after liquid–solid phase transition and demolding, a microcrystalline organic semiconductor (OSC) microstructure is obtained. During the heating stage, the melt achieves high fluidity, allowing it to fill the mold cavities (A-B), followed by system cooling (B-C-D). When the temperature at the cooler end reaches a critical point, nucleation is triggered, and crystal nuclei rapidly grow from the top of the mold, forming the patterned OSC (D-E–F). During crystallization, the temperature at the growth front increases due to the release of latent heat of crystallization (D-E) and remains constant (E–F) until crystallization is complete. After crystallization, the temperature of the OSC continues to decrease as the ambient temperature drops (F-G). It is well known that during rapid supercooled crystallization, molecules cannot form a perfectly ordered lattice due to the fast solidification process, easily leading to defects such as vacancies, dislocations, and grain boundaries [67]. Therefore, the system temperature is maintained near the melting point for post-crystallization annealing, during which the defect density is reduced to grow high-quality crystals (G-H). Finally, the system is naturally cooled to room temperature to relieve thermal stress, and the polydimethylsiloxane (PDMS) mold is removed from the substrate, yielding high-quality OSC patterns with low defect density and high uniformity. It is noteworthy that no chemicals are used in the NIL process, thereby eliminating chemical damage. This approach grants NIL prominent advantages such as low cost, high resolution (capable of reaching a few nanometers), and high throughput, making it a highly promising technology for large-area manufacturing of micro/nanostructures.

### Main NIL Techniques

Depending on the materials and energy sources used in the process, NIL can be categorized into three main types (as shown in Fig. 3a): thermal NIL, ultraviolet (UV) NIL, and electrical NIL [53]. Figure 3b demonstrates the diverse morphologies and architectures of unit components achievable by NIL, including, from left to right: disk-like units, multilayer structures, arbitrary geometric shapes, core–shell units, and multimaterial assemblies [68].

#### Thermal Nanoimprint Lithography Technology

Thermal NIL is used to pattern thermoplastic polymers and primarily addresses issues in conventional lithography, such as poor tolerance to organic materials, complex processing, and high costs [53]. This technique enables large-area, high-resolution patterning of thermoplastic polymers, resulting in OSC patterns with excellent surface quality, tunable crystallinity, and low trap density. It offers a simple, low-cost nanopatterning solution highly compatible with existing semiconductor manufacturing processes for organic optoelectronic devices.

To meet the demands of next-generation fine patterning technologies, nanoimprint lithography was first introduced in 1995 as a thermal imprinting method [45]. Thermal NIL typically involves a heating process to soften a coated thermoplastic photoresist (commonly PMMA) on a substrate, enabling it to be pressed into a pre-patterned mold under mechanical force. High pressure is maintained during the subsequent cooling phase. After mold release, the pattern is transferred to the photoresist, and any residual resist can be removed via a reactive ion etching process. Furthermore, using a similar approach, patterned structures can also be directly transferred between substrates, a technique referred to as thermal printing or thermal transfer [69–72]. Thermal NIL is a simple and highly compatible technique, making it suitable for existing materials and semiconductor manufacturing processes [72]. Zabow proposed one of the most cost-effective resists for thermal NIL: common table sugar (sucrose) [73]. The low thermal transition temperature and water solubility of sucrose greatly facilitate mold formation, demolding, and surface cleaning.

**Fig. 4:**
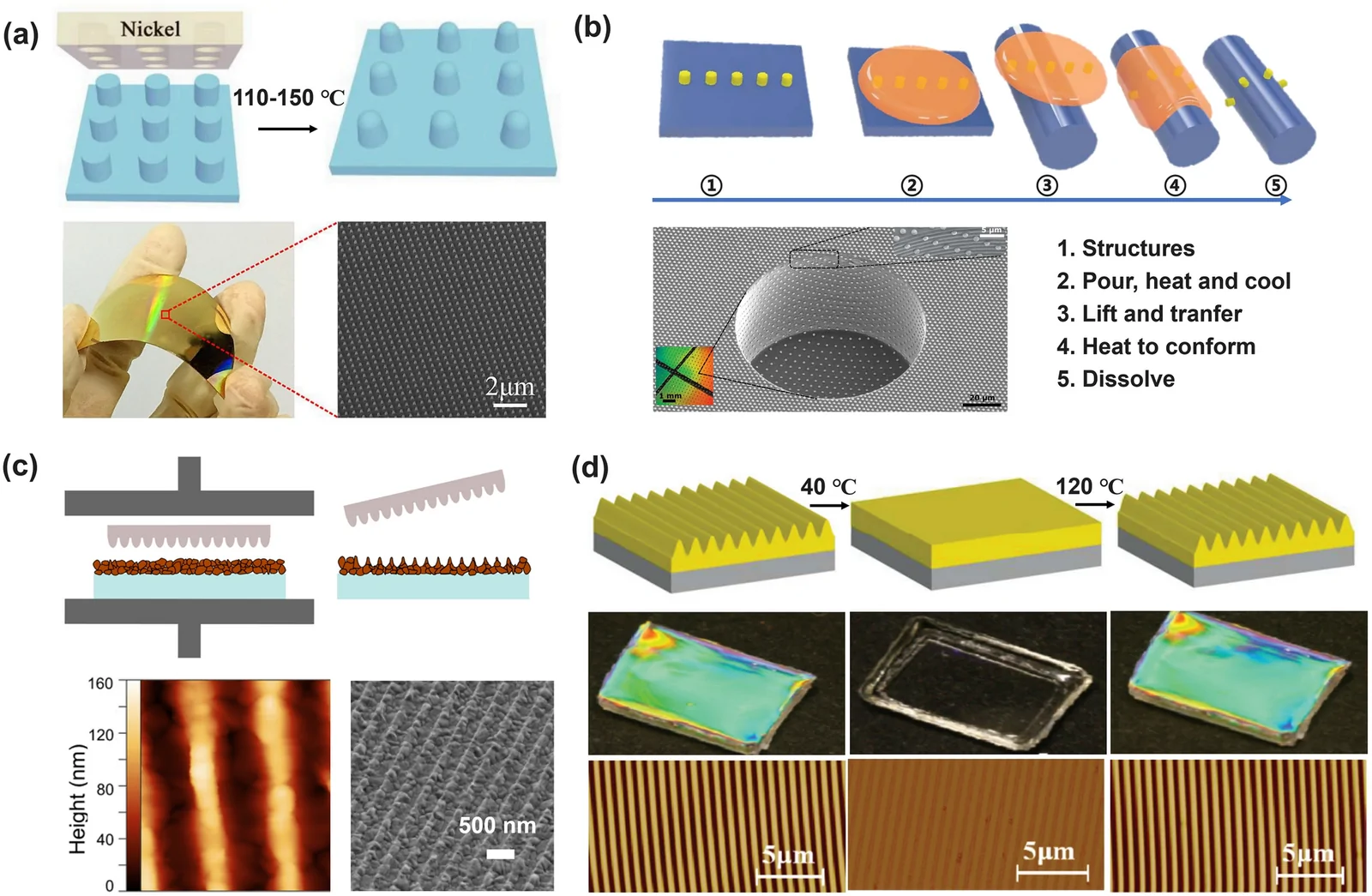
Pattern transfer achieved via thermal nanoimprinting. **a** Microscopic photograph and scanning electron microscope (SEM) image of a large-area nanostructured lattice fabricated using thermal nanoimprinting. Reproduced with permission. [75] Copyright 2020, Elsevier. **b** Process steps (1 to 5) and the transferred annular pattern onto a surface featuring 100 µm diameter, 50 µm deep holes. Reproduced with permission. [73] Copyright 2022, American Association for the Advancement of Science. **c** Patterns directly nanoimprinted onto perovskite films, as shown by AFM and SEM images. The average roughness of the grating can be as low as 4.4 nm. Reproduced with permission. [74] Copyright 2023, American Chemical Society. **d** Schematic of the pattern memory cycle in an acrylate-based polymer. The grating period and height are 834 nm and 179 nm, respectively. Reproduced with permission. [76] Copyright 2011, Wiley

Additionally, Li et al. achieved in situ control of crystallization kinetics through temperature gradient adjustment, producing OSC nanostructures with low defect density and high uniformity [56]. Compared to other lithographic techniques, this method enables the direct processing of micro- and nanostructures on flexible (Fig. 4a), non-planar (Fig. 4b), or targeted material (Fig. 4c) substrates with ease [73–75]. Conventional thermal imprinting typically uses rigid molds to minimize the effects of thermal expansion and contraction; however, rigid molds are more costly to produce, and their microstructures are more prone to damage. On the other hand, the deformable nature of soft molds can be exploited in some instances to achieve rapid shape adjustments based on pattern memory (Fig. 4d) [76].

Recently, to overcome the limitations of solution-based methods in achieving high-precision and complex patterning, Li et al. effectively addressed the trade-off between residue-free nanoimprinting and the fabrication of independent 2D complex pattern arrays through thermal NIL [56]. By leveraging precise morphological control and in situ crystallization kinetics during the molding process, thermal NIL improves both patterning resolution and crystallinity. As a chemical-free approach, it essentially eliminates chemical damage caused by solvents or developers in conventional lithography. The resulting OSC patterns exhibit excellent surface quality, tunable crystallinity, and low trap density. Valued for its flexibility, cost-effectiveness, and capability to produce high-resolution structures, thermal NIL offers a solution for programmable scaling of imprinted patterns from a single template. Studies have successfully demonstrated the fabrication of uniform, large-area nanostructured lattices over entire flexible substrates, underscoring the potential of thermal NIL for large-scale production [77]. However, the process requires precise control of temperature and pressure. Inconsistent conditions may lead to incomplete filling and difficulties in demolding. Moreover, achieving suitable glass transition temperatures (T_g_) for different imprint resists can involve extended heating and cooling cycles.

#### UV Nanoimprint Lithography Technology

Ultraviolet nanoimprint lithography (UV-NIL) is particularly suitable for integration with high-throughput R2R processes due to its room-temperature and low-pressure operation, making it one of the most promising technical routes for large-area, low-cost, and scalable manufacturing of organic optoelectronic devices [63]. It enables rapid patterning under ambient conditions, achieving fine features as small as 10 nm. This technique provides a gentler and more efficient nanofabrication solution for thermally sensitive organic materials, and equally, for delicate organic–inorganic hybrid materials such as metal halide perovskites, where preserving the crystalline integrity of the photoactive layer is paramount.

To overcome the drawbacks associated with heating/cooling cycles and high imprint pressures, Haisma et al. developed the UV-NIL process in 1996. This method uses UV-sensitive photoresists instead of thermal resists, effectively avoiding mold damage caused by heat and high pressure, thereby extending mold lifetime and reducing costs [78]. UV-NIL typically employs low-viscosity UV-curable liquid polymers, requiring mold materials transparent to ultraviolet light, such as quartz (hard mold) or PDMS (soft mold). After the mold is pressed into the photoresist, UV exposure polymerizes and solidifies the resin to form patterns [79, 80]. The rapid curing characteristic of UV-NIL enables very high throughput, and the relatively low resin viscosity allows it to fill fine mold features, achieving microscopic patterns as small as 10 nm [81]. Owing to its low cost, good conformity, low sensitivity to particulate contamination, and reduced imprint and demolding force requirements, UV-NIL has been widely adopted across multiple fields.

However, defects such as bubbles are often observed in imprinted patterns, especially in large-area applications [82]. Recently, several strategies have been proposed to address these issues, including step-and-flash NIL and R2R NIL technologies. Ahn and Guo [63] demonstrated a high-speed (7.94 mm s^−1^) and large-area (4-inch wide) R2R and roll-to-plate nanoimprinting system capable of continuously imprinting diffraction grating patterns with a linewidth of 300 nm. This system not only significantly improves production efficiency but also maintains pressure uniformity and enables successful demolding during large-area imprinting, far exceeding the throughput of conventional batch processes. Moro et al. [83] used R2R UV-NIL to transfer anti-reflective structures onto polymer films. At a feed speed of 1.8 m per minute, they produced high-performance anti-reflective films with a reflectance of 0.1% and transmittance of ~ 95% in the visible spectrum, successfully transferring anti-reflection structures to large-area polymer films with commercial-grade production efficiency and uniformity.

**Fig. 5:**
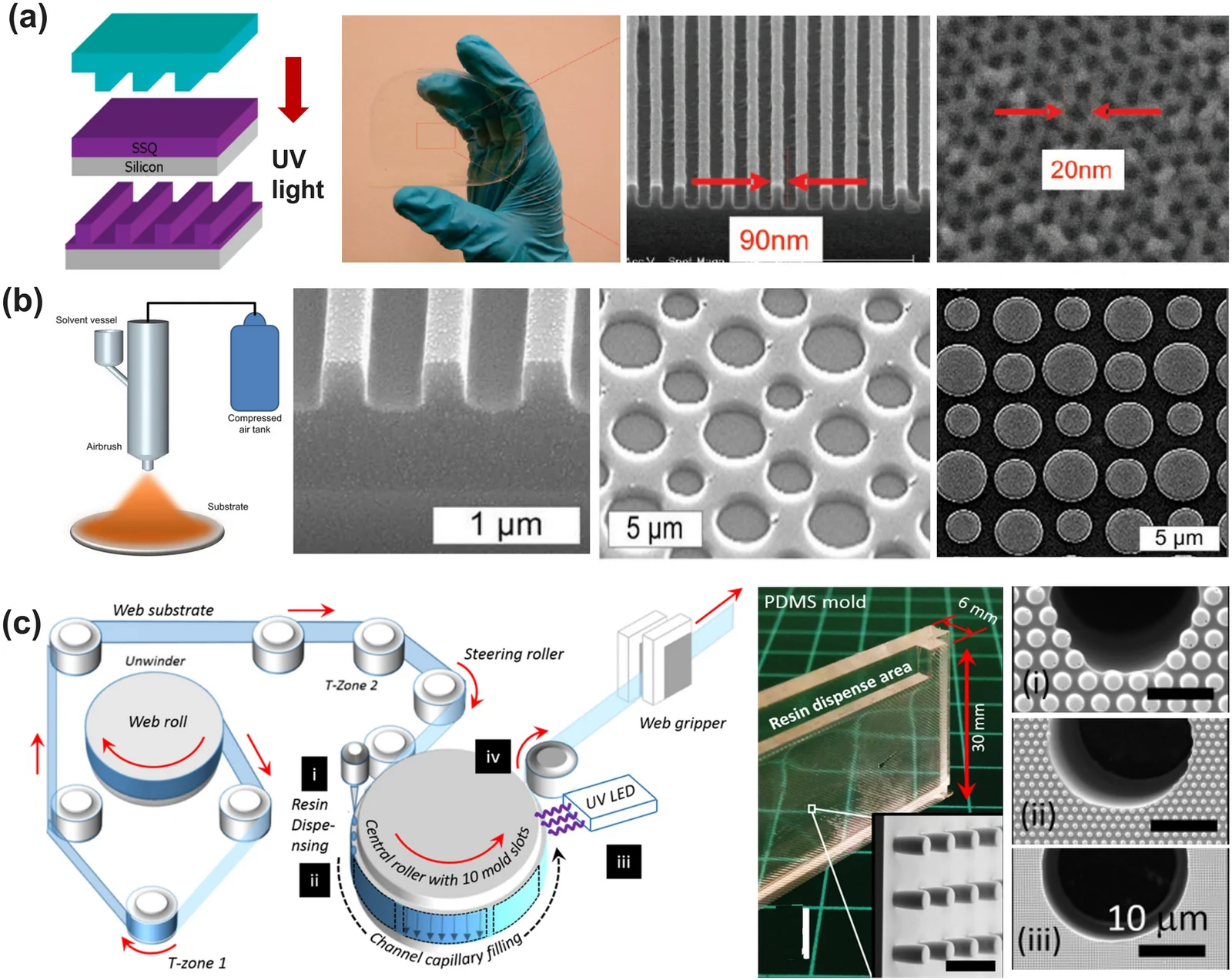
Pattern transfer in UV nanoimprinting. **a** An epoxy-siloxane-based photoresist, with UV cure times of only a few seconds, achieves resolutions up to 90 nm and 20 nm in grating and array structures. Reproduced with permission. [85] Copyright 2010, American Chemical Society. **b** Diluted polymer can be coated more rapidly via air spraying rather than spin coating, enabling continuous nanoimprinting. The right panel shows the master and printed structures. Reproduced with permission. [86] Copyright 2017, Springer Nature. **c** R2R imprint manufacturing system with high throughput up to 3000 mm^2^·min⁻^1^ and rapid UV imprinting. The right panel displays the PDMS mold used, along with an image of its microcolumn array. Reproduced with permission. [87] Copyright 2018, American Chemical Society

Although R2R processes often struggle with transferring high-aspect-ratio structures, a Japanese research team led by Hiroshi developed a breakthrough technique based on R2R UV-NIL. Using high-strength replica molds, they fabricated line-and-space patterns with an aspect ratio as high as 5 [84]. These line-and-space patterns (500 nm height, 100 nm width) were imprinted on polyester films. Photo-curable silsesquioxane resin enables a linewidth resolution of less than 100 nm (Fig. 5a), while offering rapid curing (on the order of seconds), low shrinkage, oxygen inhibition resistance, non-volatility, high modulus, easy demolding, good coating properties, and high etch resistance [85]. Beyond traditional spin-coating, a novel air spray method has been developed for silsesquioxane (Fig. 5b) [86]. The thickness of the sprayed resin film can be controlled by adjusting the polymer concentration in the solvent and the air-spraying time. Using soft molds, rapid UV curing, and optimized resist coating, continuous R2R imprinting equipment with significantly higher efficiency than thermal NIL can be designed (Fig. 5c) [87]. Multilayer nanostructures can be achieved through multiple imprinting steps—for example, first imprinting a substrate with a pattern of micron-sized pores (20 µm) and then using this substrate to imprint a PDMS micropillar mold (550 nm diameter)—greatly simplifying the fabrication of graded porous membranes. Therefore, R2R NIL is typically used to fabricate relatively larger structures or patterns. Owing to its convenience in applying mechanical pressure, this technology has been widely used to produce large-area microstructures for various applications ranging from pressure sensors and transistors to solar cells and LEDs.

The UV-NIL process can be performed at room temperature with lower mechanical force compared to thermal NIL, as the mold typically maintains a constant distance from the substrate coated with a liquid film or droplets [53]. Instant curing technology can enhance imprint speed and simplify the demolding process. However, UV-NIL resins are generally more expensive due to the use of highly customized monomers, photoacid generators, and viscosity-reducing solvents. Furthermore, these chemicals require careful handling, and volatile organic compound treatment systems are necessary. It is also essential that the substrate materials be resistant to UV exposure.

#### Electrical Nanoimprint Lithography Technology

Electrical nanoimprint lithography (E-NIL) primarily addresses the challenges of filling high-aspect-ratio structures and avoiding device damage caused by high mechanical pressure [53]. It enables the fabrication of high-aspect-ratio microstructures (up to 10:1) without mechanical pressure and allows precise control over microstructure geometry. This provides a novel solution for creating complex three-dimensional structures in high-performance sensors and optoelectronic devices.

The performance of sensors, energy storage devices, or optoelectronic components often requires microstructures with high aspect ratios. However, due to surface tension effects—especially at the nanoscale—directly patterning polymer structures with high aspect ratios using conventional NIL is difficult, which hinders the filling process [88]. Excessively high pressures can easily damage substrates, molds, and equipment. To overcome these filling challenges, Yokoo et al. [89, 90] proposed a technique using nanoelectrodes to fabricate nanostructures, known as electrochemical nanoimprint lithography. This method employs patterned conductive molds to induce electrochemical reactions on the target material surface. The process involves bringing the conductive mold into contact with the substrate so that only the protruding parts of the mold touch the substrate surface. After applying voltage, a current path forms between the mold and the substrate, and the protruding regions become sites for electrochemical reaction. Ultimately, the reacted areas on the target material replicate the pattern from the conductive mold. Through development or etching, silica patterns with a pitch of 500 nm and a thickness exceeding 5 nm can be successfully created over a uniform area exceeding 400 μm × 300 μm [89].

**Fig. 6:**
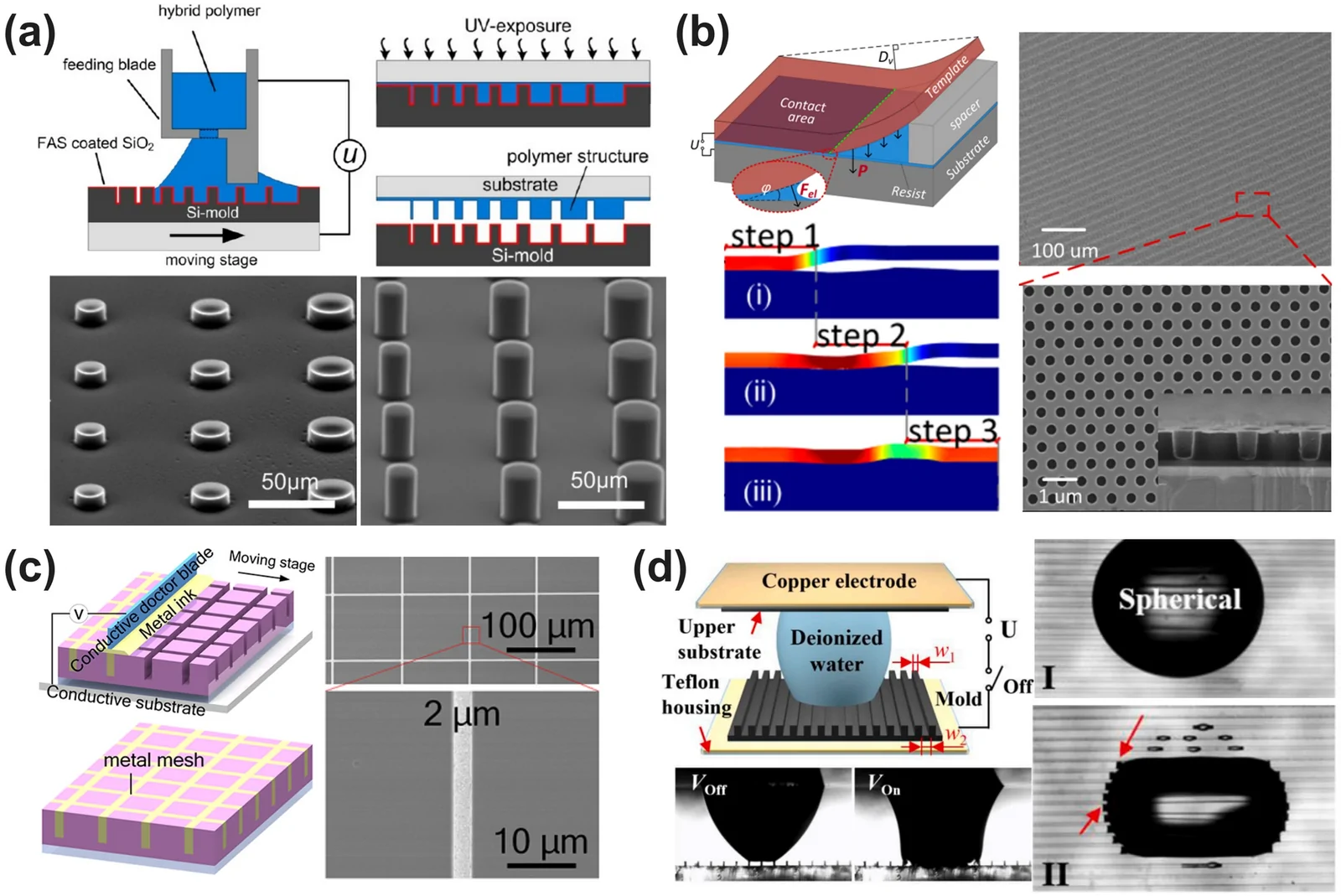
Pattern transfer in electrical nanoimprinting. **a** Continuous supply of UV-curable prepolymer onto a conductive mold with progressively graded dielectric layers; the lower panel shows height comparison of microcolumns before and after voltage application. Reproduced with permission. [91] Copyright 2014, American Chemical Society. **b** Step-release template adapted to curved substrates. (i), (ii), and (iii) illustrate sequential release steps; the right panel displays a uniform SEM moiré fringe nanopore array. Reproduced with permission. [51] Copyright 2016, American Chemical Society. **c** Metal ink filling a fine mesh via electro-wetting-assisted blade transfer, achieving embedded silver mesh structures with varying line widths. Reproduced with permission. [95] Copyright 2021, American Chemical Society. **d** Photo-resin filling mold grooves driven by an external electric field; the right image shows top views of droplets with (I) and without (II) applied external electric field. Reproduced with permission. [96] Copyright 2024, Elsevier

The research group of Shao [91] further proposed applying a properly modulated square-wave voltage between a conductive template and the substrate, which reduces the contact angle of the polymer melt within the mold cavities (Fig. 6a). The electric-field-induced electrocapillary force effectively drives the polymer to fill the nanocavities in the mold. Using electrocapillary forces independently—without mechanical assistance—makes it possible to fabricate micro/nanostructures with aspect ratios as high as 10 [91, 92]. Moreover, due to electric-field-induced surface tension, the geometry of microstructures can be specifically deformed through electric field modulation [93, 94]. For example, as shown in Fig. 6b, microlens arrays with precisely controlled curvature can be mass-produced using electrochemical NIL technology [51]. Nie et al. [95] filled fine mesh (down to submicron) and deep network microcavities with metal ink via electro-wetting-assisted blade coating, ensuring high conductivity and transparency. Based on this fully embedded metal mesh, the device’s emission intensity was enhanced more than threefold (Fig. 6c). Wen et al. [96] applied an external electric field to regulate the wetting of deionized water, ionic liquids, and high-viscosity fluids on microgrooves. Ultimately, the photosensitive resin, driven by the external electric field, filled the mold cavities, achieving conformal contact and complete replication (Fig. 6d).

E-NIL is poised to significantly benefit industrial applications due to its advantages in photoresist filling and forming microstructures into functional geometries. Electrostatic forces offer an additional benefit: Compared to mechanically driven contact processes, they typically enable uniform contact, minimizing bubble defects between the mold and the substrate by leveraging distance-dependent electrostatic effects [97]. Utilizing electrocapillary forces, Shao’s team [91] further demonstrated a versatile filling strategy for reverse NIL. During the filling process, a conductive feed blade is used to supply UV-curable polymer to the template while an appropriate voltage is applied between the feed blade and the mold to induce electrocapillary action. This allows the mold cavities to be filled with the prepolymer. The photoresist in the mold cavities can then be transferred to specific target substrates for various applications. Recently, Fan et al. [98] fabricated large-area uniformly tilted, gradient-tilted, and high-angle-tilted nanostructures by flexibly adjusting the electric field strength and angle. Compared to traditional replication, the morphology of nanoimprinted structures can be custom-controlled, offering an economical, efficient, and versatile technique for producing various large-area tilted metasurfaces. The integration of tilted nanogratings with high coupling efficiency into augmented reality displays has demonstrated excellent imaging quality.

In summary, NIL technology is a high-throughput, high-resolution, and low-cost nanofabrication method for creating functional micro/nanostructures [80]. However, several critical challenges remain. First, there is a need for high-quality molds, especially those with high aspect ratios and large areas, although direct laser writing and interference lithography offer possible strategies for mold fabrication [68]. While UV-NIL is relatively mature for various industries, electrocapillary forces enable NIL to manufacture high-aspect-ratio or deformable geometric nanostructures, meeting the demand for flexible electronic devices with diverse functions [53]. Each NIL variant has its own advantages and disadvantages, and Table 1 provides a comparative overview of the three techniques. Beyond conventional NIL, other patterning techniques based on molds or templates are often regarded as variants of NIL. These include soft lithography based on elastomeric stamps, microcontact printing for functional thin-layer patterning, and micromolding for structuring various materials. These methods are cost-effective and straightforward, demonstrating that NIL is a promising platform for fabricating high-quality OSC devices for applications in microelectronics, optoelectronics, and nanophotonics. These techniques are commonly used to generate functional structures for organic optoelectronic devices, such as OLEDs, OPVs, and OFETs.

**Table 1:** Comparison of Different Nanoimprinting Methods

Nanoimprint Method	Thermal NIL	UV NIL	Electrical NIL
Suitable Materials	Thermoplastic polymers (e.g., PMMA, sucrose), organic powders, perovskite precursors	UV-curable liquid polymers (e.g., silsesquioxane resin), photoresists	UV-curable polymers, metal inks, high-viscosity fluids, functional materials
Key Requirements/Parameters	Requires heating above glass transition/melting point (T_g_/T_m_), high mechanical pressure, controlled cooling cycles	Room temperature operation, low mechanical pressure, UV exposure for curing, transparent mold/substrate required	Applied voltage/electric field (electrocapillary force), low or no mechanical pressure, conductive mold/electrodes
Typical Results/Performance	High-resolution patterns (20–100 nm), large-area uniform nanostructures on flexible substrates, tunable crystallinity, low defect density	High resolution (~ 10 nm), high-throughput R2R compatible, rapid curing (seconds), excellent pattern conformity over large areas	Enables high-aspect-ratio structures (up to 10:1), precise control over microstructure geometry (e.g., microlens curvature), uniform filling minimizing bubbles
Specific Applications	Direct patterning of OSC microcrystals, large-area flexible electrode fabrication for OLEDs/OPVs, residue-free complex 2D arrays	High-throughput manufacturing of large-area flexible OLEDs and OPVs, anti-reflective films, embedded electrode grids, integrated with R2R processes	Fabrication of high-aspect-ratio OFET electrodes, tunable metasurfaces (tilted nanostructures), embedded conductive meshes for transparent electrodes, complex 3D structures
Advantages	High flexibility in materials and dimensions, direct processing on flexible/non-planar substrates, cost-effective resists available (e.g., sucrose)	High alignment accuracy, rapid curing, suitable for R2R processing, gentle process for thermally sensitive materials	Low contact pressure protects master/substrate, enables patterning of difficult-to-fill high-aspect-ratio cavities, versatile for deformable geometries
Challenges	Requires precise temperature/pressure control, potential for incomplete filling/demolding, extended thermal cycles, rigid master molds prone to damage	Requires UV-transparent masters, resins can be expensive/customized, potential for bubble defects, substrate must resist UV exposure	Limited material compatibility (requires responsive materials), process complexity higher, resolution may be lower compared to thermal/UV NIL

### Mechanical Considerations and Mold Materials in NIL

The efficacy of nanoimprint lithography, as a mechanical forming process at the micro- and nanoscale, fundamentally depends on the precise control of interfacial mechanics to ensure pattern fidelity, uniformity, and high yield across large areas [99]. Paramount challenges inherent to this contact-based method include achieving and maintaining a uniform pressure distribution over the imprint area, preventing the collapse of delicate high-aspect-ratio features during the critical demolding step, and minimizing pervasive defects such as incomplete cavity filling and resist adhesion to the mold [100].

The selection and engineering of the mold are central to addressing these challenges. The choice of an appropriate mold material is dictated by the specific NIL technique and its application demands. For the most demanding applications in terms of precision and durability, rigid materials are indispensable. Fused quartz is essential for ultraviolet nanoimprint lithography due to its optical transparency, while silicon masters offer atomic-level smoothness for nanofabrication. In industrial R2R nanoimprint lithography, electrodeposited nickel stamps stand as the workhorse material due to their outstanding mechanical durability and replication fidelity [101]. For scenarios requiring a balance between flexibility and precision, elastomers like polyurethanes or fluorinated elastomers offer a higher elastic modulus than PDMS, enabling better pattern fidelity in high-resolution ultraviolet nanoimprint lithography while retaining sufficient conformability for non-planar substrates. When high rigidity, exceptional thermal stability, and minimal shrinkage are required, thermosetting polymers such as epoxy-based resins or inorganic–organic hybrid materials like organically modified ceramics serve as excellent choices for durable daughter molds or high-temperature thermal nanoimprint lithography processes [101, 102].

Beyond the bulk material, the mold’s surface properties play a decisive role. Surface chemical modifications, such as the application of anti-adhesion layers (e.g., fluorinated silanes or diamond-like carbon films), are widely used to reduce interfacial adhesive forces drastically [103]. This treatment is critical for facilitating clean demolding, protecting delicate nanostructures from damage, extending mold lifetime, and thereby enhancing process robustness and yield. This aspect of surface engineering significantly broadens the applicability of NIL by enabling the patterning of more challenging material systems and complex device architectures with higher fidelity [103].

A frequently underappreciated yet critical parameter stemming from direct mechanical contact is the surface roughness replicated at the mold-polymer interface. This nanoscale texture, if excessive, can introduce deleterious light scattering in photonic devices, act as charge-trapping sites, degrade electronic performance, and mechanically complicate the demolding process [104]. Its origins are multifaceted, arising from imperfections in the master mold fabrication, flow instabilities of the polymer resist during filling, and specific interfacial interactions. Achieving ultra-smooth interfaces with sub-nanometer roughness typically requires starting with superpolished master molds fabricated via techniques such as electron-beam lithography and dry etching, coupled with meticulously optimized imprint parameters—such as temperature, pressure, and duration—to promote a homogeneous, viscous flow regime that faithfully replicates the mold geometry rather than its surface texture [101].

The applicability of NIL is further evidenced by its successful expansion beyond organic material systems. In recent years, its use has been successfully extended to perovskites, metal oxides, and 2D materials [105–107]. For instance, low-temperature UV-NIL or electrochemical NIL can pattern micro- and nanostructures on perovskite layers without damaging the photoactive material, thereby enhancing light-harvesting efficiency and device stability [108]. Nanopatterning of metal oxides (e.g., ZnO, TiO₂) via thermal NIL or sol–gel-assisted NIL enables the construction of high-performance transparent electrodes or electron transport layers. For 2D materials such as graphene and transition-metal dichalcogenides, NIL can fabricate high-quality electrodes or induce an aligned arrangement, boosting device performance. Moreover, in terms of process integration and complexity, advances such as the self-aligned sequential multilayer nanoimprinting demonstrated by Oh et al. highlight the potential for ultra-low-cost, large-area multilayer patterning [109]. This capability is particularly promising for applications such as full-color displays and integrated optoelectronics, while the low-temperature, low-pressure nature of these processes ensures compatibility with flexible substrates.

When contrasted with alternative non-contact or minimally-contact patterning methods, such as capillary force lithography or electrohydrodynamic patterning, which utilize surface tension or electric fields to structure materials, nanoimprint lithography offers distinct advantages and trade-offs [53]. While techniques relying on self-organization or field-driven assembly can address some contact-related issues, NIL offers unparalleled advantages in deterministic pattern fidelity, dimensional control across a wide range of feature sizes, and compatibility with a broader range of material viscosities and compositions [54]. The direct mechanical contact intrinsic to NIL, though demanding careful management of forces and interfaces, is precisely what enables its high-resolution replication and robust large-area scalability. Ultimately, the choice of patterning technique depends on the specific application requirements. For the fabrication of high-performance organic optoelectronic devices, where precise micro- and nanoscale structuring over large, flexible areas is paramount, NIL emerges as a uniquely powerful and versatile platform [53, 106]. Its continued evolution, driven by advances in mold engineering, material science, and process control, solidifies its pivotal role in bridging the gap between nanoscale design and macroscopic functional device manufacturing.

## Application of Nanoimprint Lithography in Organic Optoelectronic Devices

Nanostructures—such as nanopores, nanotips, nanowires, and nanomeshes—have been widely recognized as key components in future manufacturing fields, including solar cells, optoelectronic devices, nanoscale sensors and actuators, high-density data storage, biological devices, and microfluidics [110, 111]. Owing to its versatility, high patterning resolution, tunable crystallinity, and ultra-low defect density, NIL technology can flexibly fabricate structures of various sizes and shapes on different substrate materials (e.g., glass, semiconductors, metals, and plastics) [112]. Currently, NIL is one of the most successful nanofabrication techniques in commercial applications. Due to its cost-effectiveness and mass-production capability, NIL continues to be widely explored in both academic and industrial settings for use in OLEDs, OPVs, and OFETs [113, 114]. This section briefly reviews the main contributions of NIL in the field of organic optoelectronics and summarizes recent advances and ongoing challenges.

### Organic Light-Emitting Diodes

#### Efficiency Enhancement and Flexibility Fabrication

**Fig. 7:**
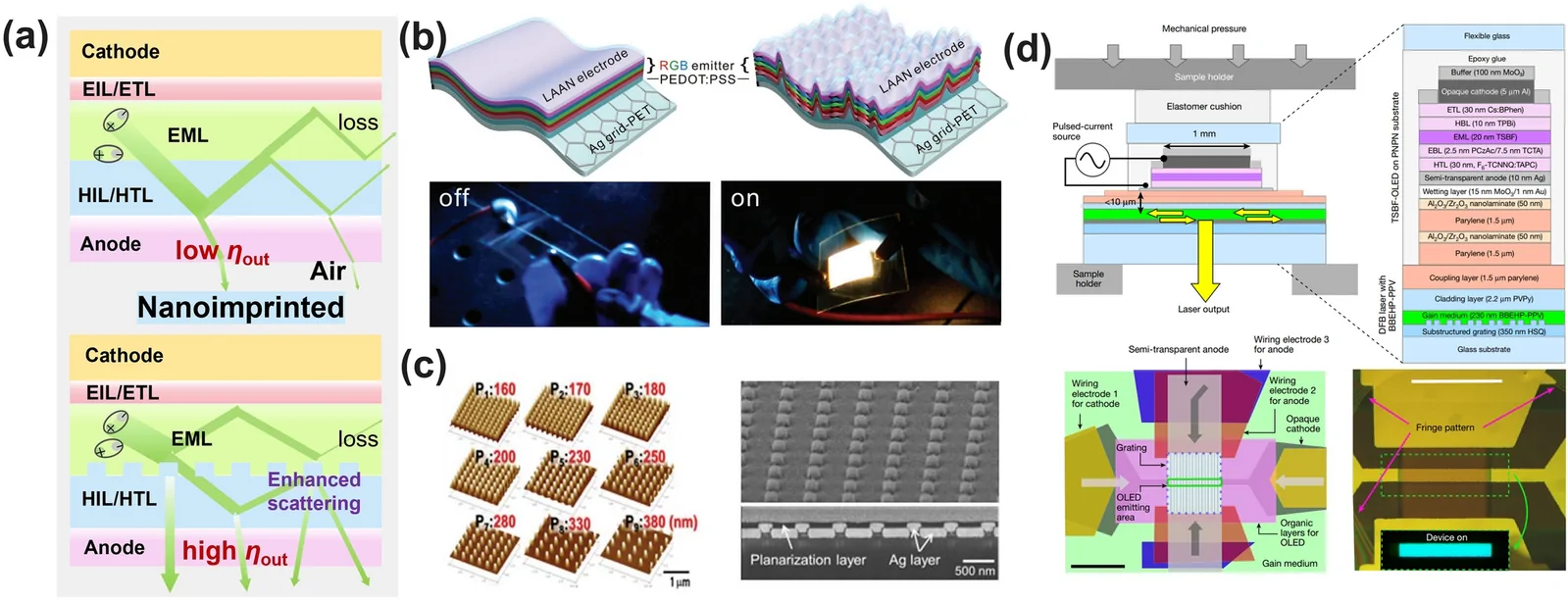
Application of nanoimprinting in organic light-emitting diodes. **a** Schematic of the performance enhancement mechanism in an OLED achieved via NIL. **b** Schematic of an OLED on a plastic substrate with and without an internal moth-eye external coupling structure. Below are photographs of the OLED in off and on states. Reproduced with permission. [127] Copyright 2018, Wiley. **c** Atomic force microscopy image of a superlens showing nanocolumn arrays with varying spacings (P1 = 160 nm to P9 = 380 nm) and constant diameter (80 nm) and height (80 nm). The right panel shows SEM images of the Ag superlens prepared via nanoimprint lithography: an overview (top) and cross-section (bottom). Reproduced with permission. [64] Copyright 2020, American Association for the Advancement of Science. **d** Schematic cross-section and top view of the electrically driven laser; the bottom panel shows a microscopic image of the electrically driven laser. Reproduced with permission. [65] Copyright 2023, Springer Nature

OLEDs have achieved remarkable commercial success in panel displays for smartphones and televisions, as well as in solid-state lighting, owing to their flexibility, ultra-thin design, high contrast ratio, and wide viewing angle [115–118]. Extensive research has confirmed that micro- and nanostructures can effectively enhance the light extraction capability of OLEDs [119–124]. NIL, with its cost-effectiveness and potential for mass production, offers a promising route to integrate such structures into OLED manufacturing, addressing key challenges such as low light extraction efficiency and difficulties in fabricating high-performance, flexible electrodes [53]. Figure 7a illustrates the mechanism for performance enhancement in NIL-fabricated OLEDs, with detailed performance comparisons provided in Table 2.

**Table 2:** Performance Enhancement of OLEDs via NIL Technology [64, 65, 126–139]

Device Structure	Imprinted Layer	Period	Height	Enhancement		References
glass substrate/SU-8/Ag/Au/MoO_3_/NPB/CBP: (IrBT)_2_ (acac)/TPBi/Ca/ Ag–Al	Au film	250 nm	70 nm	30% enhancement of current efficiency and luminance		[126]
Substrate&Ag grid/PEDOT:PSS/EML/Ag/NPB	PEDOT: PSS	300 nm	50 nm	external quantum efficiency of 72.4% and power efficiency of 168.5 lm W^−1^ with a color-rendering index over 84		[127]
ITO/EML/Ag	Ag film	160–380 nm	80 nm	twofold increase in luminescence efficiency and a higher color purity than color-filtered white-light OLEDS and an ultra-high pixel density of 10,000 PPI		[64]
Al/Cs:BPhen/TPBi/TSBF/TCTA/Ag/MoO3/Au	HSQ	-	-	achieve spatial separation between charge injection and laser operation, thereby significantly reducing losses		[65]
ITO/HATCN/NPB/mCP/Pt(II)No.2/BTPV-eC9/PO-T2T/LiF/Al	Pt(II) complex	-	-	a remarkable radiance of 39.97 W sr^−1^ m^−2^ and an exceptional EQE of 2.24% (1.94 ± 0.18%) with an emission peak wavelength of 925 nm		[128]
Cu mesh/PEDOT:PSS/MEH-PPV/LiF/Al	Au, Al, and Cu	700 nm	200 nm	high optical transmittance in the visible range of the electromagnetic spectrum as well as excellent electrical conductivity		[129]
anode/PEDOT:PSS/SY /CaAl/Ag	Ag film	400 nm	20 nm	twofold electroluminescence		[130]
Au/TCTA/BPhen/LiF/Al	Au film	200 nm	15 nm	1.57-fold higher external-quantum-efficiency and light-extraction-efficiency, ambient-light absorption 2.5-fold higher, fivefold higher contrast, 1.86-fold higher normal-view-brightness, uniform color over all emission angles		[131]
ITO/PEDOT:PSS/TAPC/EML/B3PYMPM/LiF/Al	PEDOT: PSS	250 nm	80 nm	the resulting efficiency is over 2 times that of a conventional OLED		[132]
ITO/ZnO/interlayer/EML/Al	ZnO film	400 nm	120 nm	an external quantum efficiency of 42.4% and a power efficiency of 85.4 lm W^−1^		[133]
ITO/HATCN/1-bis[4-[N,N-di(4-tolyl)amino] phenyl]-cyclohexane/FIrpic/(26DCzPPy): FIrpic/BmPyPB/BmPyPB:Li/LiF/Al	Film DC	10–20 μm	-	the external quantum efficiency increased by a factor of 1.35, power efficiency increased by a factor of 1.86, achieve a high color-rendering index of 84.3		[134]
ITO/PEDOT:PSS/TAPC/mCP:10 wt% FIrpic/TmPyPB/LiF/Al	PEDOT: PSS	500–600 nm	40 nm	a more than 35% efficiency enhancement		[135]
substrate/PVA/SU-8/Au/MoO_3_/NPB/CBP:(Ir(MDQ)_2_(acac)/TPBi/Ca/Ag	Au film	280–330 nm	55 nm	the current efficiency and luminance have been enhanced 45.3% and 18.1%, respectively		[136]
ITO/NPB/Alq_3_/Bphen/LiF/Al	PDMS	-	-	the external quantum efficiencies were enhanced by 20.9%		[137]
substrate/Al/Ag/HAT-CN/TAPC/HAT-CN/TAPC/HAT-CN /NPB/Bebq_2_:Ir(mphmq)_2_(tmd)/Bphen/LiF/Al/Ag/MoO_3_	IRMLA	10–20 μm	4 μm	an impressive 58% enhancement in external quantum efficiency		[138]
ITO/HAT-CN/TAPC/TCTA/CBP:Ir(ppy)_3_/TmPyPB/LiF/Al	PC:PS, PVDF:PS	-	-	EQE of two OLEDs incorporating these structures is improved by 69.2 and 61.7%, the viewing angle ratio is increased by 10.6% and 7.7%, respectively		[139]

A primary strategy involves the direct nanoimprinting of functional electrode layers. Since the introduction of NIL technology, Chou et al. [125] have pioneered its application in organic materials, utilizing integrated micro/nanostructures to manipulate the light field in organic optoelectronic devices effectively. Both small-molecule and polymer submicron structures have been successfully fabricated using NIL. Following this principle, Ma et al. [126] employed polymer assisted thermal NIL to imprint ultra-thin metal films directly, evaporating the metal onto uncured photopolymers. This approach eliminated non-uniform evaporation caused by nucleation and growth variations on the facets, edges, and corners of the nanostructures, significantly enhancing the performance of OLEDs fabricated with nanoimprinted electrodes. A primary challenge for this approach is ensuring the imprinted metal structures maintain both high optical transparency and low electrical resistance.

A more advanced strategy focuses on creating integrated nanostructures for flexible, transparent electrodes. In recent years, transparent flexible OLEDs have garnered significant attention due to their potential for use in active-matrix displays for wearable, portable, or foldable electronics, as well as high-aspect-ratio microdisplays. Xiang’s group [127] leveraged the capabilities of NIL to successfully fabricate centimeter-scale flexible OLEDs based on embedded silver grid electrodes on PET substrates (Fig. 7b). They used UV-assisted NIL with a nickel mold to imprint a hexagonal silver grid, followed by thermal NIL to form a nanostructured PEDOT: PSS film on the Ag grid-PET surface, further enhancing conductivity. This structure achieved over 70% device transparency and highly efficient warm white emission on both sides. The key feature is the transparent metal-dielectric composite electrode patterned with moth-eye nanostructures, enabling broadband, angle-independent light outcoupling and achieving record values of 72.4% external quantum efficiency and 168.5 lm W⁻^1^ power efficiency [127]. This work demonstrates the potential of multistep NIL processes for complex device architectures; however, challenges remain in terms of process alignment yield and long-term mechanical stability under repeated bending.

While the performance of NIL-fabricated OLEDs is impressive (Table 2), it is important to note that comparable efficiencies have also been achieved with other patterning or non-patterned device-engineering methods. The distinctive advantage of NIL lies in its ability to deliver high efficiency, transparency, and flexibility concurrently through a scalable patterning technique, addressing a combination of requirements critical to next-generation displays.

#### Ultra-High-Density Displays

OLEDs are widely used in high-resolution, large-area televisions and handheld displays for smartphones and tablets, where viewing distances are relatively large, and typical pixel densities range from a few hundred to several thousand pixels per inch (PPI). However, for near-eye microdisplays (e.g., in virtual and augmented reality applications), pixel densities of several thousand PPI are required, which pose significant challenges for current display technologies. Here, NIL provides a potential pathway. Joo et al. [64] developed a full-color, high-brightness OLED design based on an engineered metasurface that serves as a tunable back reflector. By incorporating nanoimprinted metasurface mirrors, they achieved a complete architectural redesign of the OLED display (Fig. 7c). This new architecture demonstrated the feasibility of fabricating devices with ultra-high-definition pixel densities (> 10,000 PPI) using scalable nanofabrication, a crucial capability for emerging display applications. However, the mass production of such metasurface-based displays would require overcoming challenges in fabricating large-area, defect-free master molds and in achieving high-yield imprinting of complex optical structures.

#### Novel Device Architectures

Beyond conventional lighting and displays, NIL enables the fabrication of novel OLED-based device concepts by patterning specialized functional layers. For instance, to address challenges in electrically driven organic lasers, Yoshida’s team [65] developed an integrated device structure that spatially separates the charge injection and lasing regions. This design significantly reduces losses, enabling an electrically driven organic laser diode pumped by an integrated OLED (Fig. 7d). They used a nanoimprinted grating as an etch mask for reactive ion etching, transferring the pattern into an underlying cladding layer and controlling the grating depth by adjusting the etch time. The fabricated lasers showed uniform thresholds with a production yield of approximately 60%. This work illustrates how NIL can achieve precise patterning for integrated photonic devices, addressing a significant challenge in organic optoelectronics. The reported yield figure, however, underscores the ongoing challenge of process control and reproducibility in the fabrication of such complex devices.

Similarly, NIL facilitates advanced material integration for spectral engineering. As the theoretical maximum EQE of OLEDs is determined by photoluminescence efficiency and charge balance, while the outcoupling efficiency varies significantly with the refractive indices of the OLED multilayer stack, Hung et al. [128] utilized the advantages of self-assembly and transfer printing, combined with short-range interfacial energy transfer, to imprint a layer of specific near-infrared fluorescent dye BTP-eC9 onto a thin film of Pt(II) complex. OLEDs based on this imprinted bilayer structure harnessed most of the Pt(II) complex phosphorescence, achieving a leap in performance for near-infrared OLEDs. This approach showcases NIL’s potential to integrate dissimilar materials with nanoscale precision to manipulate energy-transfer processes. A key consideration for such strategies is ensuring the stability and integrity of the delicate imprinted interface under electrical operation.

In summary, nanoimprint lithography enables the direct mechanical patterning of organic layers with desired dimensions and shapes, which is advantageous for overcoming limitations related to photon diffraction and electron scattering, and for allowing the fabrication of nanoscale patterns over large areas [113]. Leveraging this advantage, NIL could play an increasingly important role in the development of advanced solid-state lighting and displays. Incorporating such nanostructures into OLEDs offers a route toward low-cost manufacturing with enhanced light efficiency, contributing to the development of next-generation display technologies. Nevertheless, transitioning these laboratory demonstrations to commercial products requires further progress in throughput, cost reduction, and the reliable integration of NIL processes with existing OLED manufacturing lines.

### Organic Photovoltaics

**Fig. 8:**
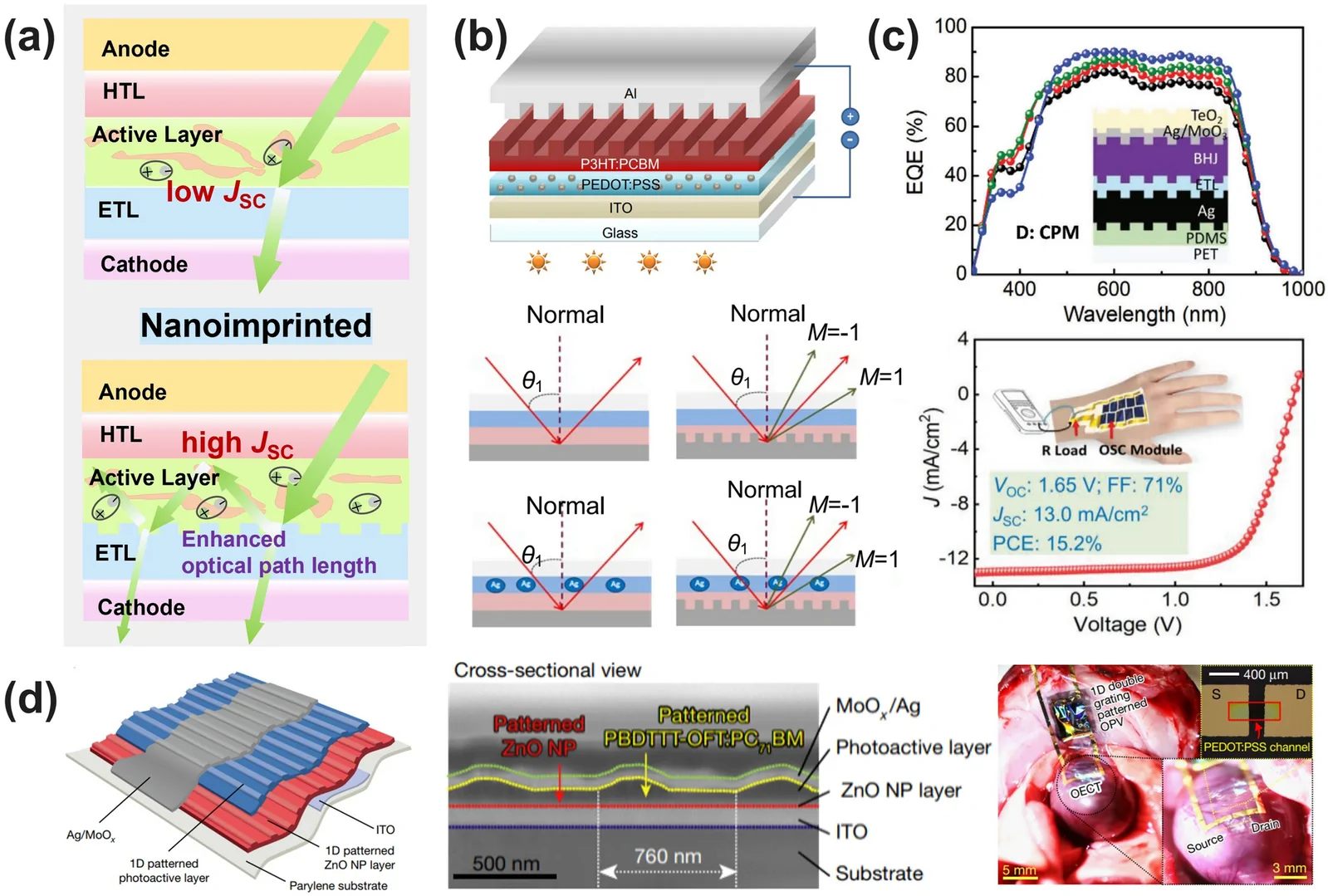
Applications of nanoimprinting in OPVs. **a** Schematic of the performance enhancement mechanism in an OPV achieved via NIL. **b** Schematic of an organic solar cell device, with a comparison below of four electrode architectures: planar Al, Al grating, PEDOT:PSS embedded with Ag nanodots, and a hybrid structure combining PEDOT:PSS/Ag NDs with an Al grating. Reproduced with permission. [143] Copyright 2019, Springer Nature. **c** Comparison of the EQE spectra for the corresponding devices, with the inset depicting the pillar-based microcavity structure; the bottom panel presents the current density–voltage characteristics of the flexible OSC module. Reproduced with permission. [147] Copyright 2024, Wiley. **d** Design and cross-sectional SEM image of a high-performance ultra-flexible OPV with a dual-grating pattern; the right image shows a photo of the self-powered integrated electronic device attached to a finger. Reproduced with permission. [9] Copyright 2018, Springer Nature

Solar energy stands as one of the most crucial future renewable resources, yet its development still faces technical bottlenecks in high-efficiency and low-cost manufacturing [140]. The widespread application of photonic structures (such as photonic crystals and transparent electrodes) in optoelectronic devices offers new avenues to address these challenges [141]. NIL has emerged as a compelling patterning platform to address this need, enabling the scalable integration of light-management micro/nanostructures. Its applicability spans several key design strategies, each with distinct mechanisms and associated integration considerations. A schematic illustrating the performance enhancement mechanism for NIL-fabricated OPVs is shown in Fig. 8a. Multiple research groups have successfully utilized this technology for patterning nanostructures in solar cells, with a quantitative performance comparison summarized in Table 3 [9, 45, 142–155].

**Table 3:** Performance Enhancement of OPVs via NIL Technology [9, 45, 142–155]

Device Structure	Imprinted Layer	Period	Height	Enhancement	References
ITO/ZnO/PBDTTT-OFT:PC_71_BM/MoOx/Ag	Photoactive/ZnO layer	760 nm	66–116 nm/10–20 nm	giving a high power-conversion efficiency that reached 10.5 percent and resulted in a high power-per-weight value of 11.46 watts per gram	[9]
-	PMMA	120 nm	100 nm	-	[45]
ITO/PEDOT:PSS/P3HT-PCBM/Al/Ag	Ag film	500 nm	30 nm	EQE enhancements up to 1.7 are shown in the weakly absorbing spectral range	[142]
Al grating/P3HT:PCBM/PEDOT:PSS-Ag NDs-70/ITO	P3HT:PCBM	-	-	photovoltaic conversion efficiency increased to 3.32%–3.59% (representing an improvement of 11%-20%)	[143]
ITO/PEDOT:PSS/PTB7-Th:PC71BM/ZnO/PEDOT:PSS/PTB7-Th:PC71BM/ZnO/Al	-	600 nm	551 nm	after applying the light-management structure to a packaged tandem cell with an efficiency of 8.7%, its conversion efficiency increased to 9.5%	[144]
PET/AgNWs/ZnO:Cs_2_CO_3_/D18:Y6:PC71BM/MoO_3_/Ag	Ag film	-	-	energy conversion efficiency increased by 8.7% compared to the control group, reaching an industry-leading level of 18.1%	[145]
glass/PEDOT:PSS/PTB7:PCBM/LiF/Al	glass	300 nm	280 nm	light absorption increased by up to 19% compared to thick-film devices, while short-circuit current improved by 14% over corresponding thick-film devices	[146]
PET/ PDMS/Ag/ETL/BHJ/MoO_3_/Ag/TeO_2_	PDMS	-	-	device efficiency increased from 17.26% to 19.11%, with accelerated aging tests demonstrating an extrapolated lifespan of approximately 23 years	[147]
ITO/TDPTD/PCBM/Al	TDPTD	510/700 nm	200 nm	the rate of increase of JSC exceeds the rate of increase in the interfacial area in corresponding devices	[148]
-	Pc5c	30 μm	50 nm	-	[149]
-	PEDOT: PSS	200 nm	22 nm	surface energy anisotropy induced by the crystallinity creates a local minimum in the free energy	[150]
ZnO/P3HT:ICBA/MoO_3_/Ag	active layer	650 nm	100 nm	increase of short-circuit current density (J_sc_) from 10.9 to 13.32 mA/cm^2^, enhancement percentage of 22.2%	[151]
ITO/ZnO NPs/donor polymer:acceptor/MoO_3_/Ag	ZnO/BHJ film	760 nm	35/55 nm	increased the light absorption and the power conversion efficiency by up to 32.5%	[152]
ITO/ZnO/ PTB7:PC71BM /MoO_3_/Ag	ZnO layer	760 nm	25 nm	the PCE have a 21% improvement compared to OPVs without the pattern	[153]
ITO/PEDOT:PSS-Nb_2_C/PM6:BTP-eC9:L8-BO/BCP/Au/Ag	PDMS	900 nm	500–600 nm	achieving a record-high PCE of 15.2%, a high AVT of 32%, an impressive light utilization efficiency of 4.86%, and a favorable color-rendering index of 82	[154]
ITO/ZnO/BHJ/MoO_3_/Ag	PDMS	300 nm	170 nm	an increase in device efficiency from 17.26% to 19.11%	[147]
ITO/Ph-4PACz/D18/L8-BO/PNDIT-F3N/Ag	D18/L8-BO	1000 nm	8.7–29 nm	exhibit record-setting metrics: 20.08% power conversion efficiency, 27.34 mA cm^−2^ short-circuit current density, and 80.34% fill factor	[155]

#### Nanoimprinted Electrodes for Precise Light-Management

A significant strategy involves using NIL to pattern electrodes or charge transport layers, thereby improving both optical and electrical properties. Regarding electrode optimization, Van de Groep et al. [142] employed a bilayer PDMS stamp NIL technique to achieve reliable transfer of high-resolution nanopatterns in a fast, simple, and inexpensive manner. Using a P3HT-PCBM model system, they fabricated silver nanowire networks covering centimeter-scale areas on glass substrates. By precisely controlling the position, size, and spacing of nanowires, combined with spin-coating sacrificial PMMA layers and silica sol–gel layers, they formed highly uniform wire networks. This top–down NIL process produced networks with a sheet resistance as low as 8.7 Ω sq^−1^, an average transmittance of 87%, and up to a 1.7-fold enhancement in quantum efficiency in the weak absorption region, outperforming conventional ITO. This demonstrates NIL’s capability for deterministic patterning of alternative transparent electrodes. A key challenge for this approach is to transfer the process to flexible substrates while maintaining the nanowire network’s conductivity and mechanical adhesion.

Beyond continuous nanowires, NIL can create plasmonic nanostructures for advanced light trapping. Putnin et al. [143] innovatively combined imprinted aluminum gratings with silver nanodisks embedded in the PEDOT: PSS layer. This configuration utilized multiple surface plasmon resonances to enhance device efficiency by 11%-20% compared to planar counterparts (Fig. 8b). While effective for boosting light absorption, such plasmonic-electrode designs must carefully manage potential parasitic absorption losses in the metal and ensure compatibility with the solution-processing of subsequent organic layers.

#### Large-Area Fabrication

Research on large-area organic photovoltaics (OPVs) is crucial for commercialization, requiring a shift from lab-scale “hero devices” to larger, stable modules for applications like building-integrated PV and flexible electronics. Leveraging OPVs’ solution processability, flexibility, and semi-transparency enables applications in portable power and IoT devices. R2R processing drives low-cost, high-throughput manufacturing, supporting industrial advancement [156–158]. Notably, NIL is well-suited to R2R processes. It can incorporate micro- and nanophotonic structures (e.g., gratings and nanopillars) to enhance light absorption via scattering and trapping, offering a promising strategy for improving the performance of large-area OPVs.

A key direction is the integration of NIL-patterned light-management structures with high-throughput R2R platforms. Mayer et al. [144] developed diffractive nanostructures that optimize the redistribution of light wavelength and propagation angle between two sub-cells, increasing tandem cell efficiency from 8.7% to 9.5%. These optical structures are fully compatible with R2R production, offering a cost-effective solution for printed photovoltaics. However, maintaining nanoscale pattern fidelity and optical performance over continuous, high-speed R2R imprinting remains a significant engineering challenge.

Similarly, NIL is used to tailor electrode morphologies on flexible substrates in a scalable manner. Hou et al. [145] introduced lateral bud-like electrode structures via thermal NIL on silver nanowires, boosting the efficiency of a flexible OPV to 18.1% by reducing plasmonic loss and enhancing light in-coupling. This demonstrates the potential of imprinting to modify commercially available transparent conductors. The challenge lies in achieving uniform patterning over the entire web width and ensuring the durability of the imprinted nanostructures during R2R handling and device operation.

Furthermore, NIL enables the replication of master templates for broadband photon management. Chowdhury et al. [146] fabricated large-area nanogroove templates using laser interference lithography and etching and then used them to create anti-reflective structures via NIL, achieving a 19% enhancement in optical absorption. This highlights the role of NIL as a replication tool for transferring pre-defined, complex optical designs. The scalability of this approach ultimately depends on the cost and availability of the master templates, as well as the longevity of the replication molds in production.

#### Flexible Integration and Applications

Flexible integrated systems represent a critical frontier for OPVs, and NIL offers a pathway to incorporate performance-enhancing nanostructures into such platforms. In flexible integrated systems, Fan et al. [147], based on a pillar microcavity structure for quaternary organic solar cells (Fig. 8c), successfully modulated the molecular crystallinity and nanomorphology of NFA-based BHJ films by incorporating naphthoquinone n-type doping into a ternary BHJ system. This simultaneously optimized molecular crystallinity, phase morphology, light-harvesting efficiency, and charge carrier transport properties, culminating in a notable power conversion efficiency of 19.11%. After inserting a thin C70 layer between the BHJ and MoO_3_ layers, the device lifetime, extrapolated from accelerated aging tests, reached approximately 23 years. More impressively, they successfully fabricated a monolithic 5 × 2 array flexible OSC module with a total area of 5 cm^2^, achieving a high efficiency of 15.2% with excellent stability. This work demonstrates the use of NIL to fabricate sophisticated optical cavities for high-efficiency, stable, and flexible modules. A significant challenge for commercial translation is scaling this process to even larger areas while maintaining high yield and performance uniformity.

With the growing demand for self-powered devices in next-generation biomedical applications, flexible photovoltaics show great potential in the biomedical field [159, 160]. Park et al. [9] integrated an organic electrochemical transistor with an organic photovoltaic power source on a one-micrometer-thick ultra-flexible substrate. Using NIL, they formed a nanograting morphology with a 760-nm period on the charge transport layer (Fig. 8d), achieving a power conversion efficiency of 10.5% and a high gravimetric power of 11.46 W g⁻^1^. This self-powered system exhibited a transconductance of 0.8 mS and a response speed exceeding 1 kHz under physiological conditions, with a signal-to-noise ratio of 40.02 dB for cardiac signal detection, providing an innovative solution for next-generation wearable medical electronics. This example highlights NIL’s role in enabling multifunctional, miniaturized bio-electronics. However, ensuring the long-term biocompatibility and environmental stability of such ultra-flexible, patterned devices in vivo remains a key hurdle.

These findings demonstrate that nanoimprint lithography, through precise control of nanostructures, can effectively address the problem of insufficient light absorption in OPV devices and aid in the development of advanced device architectures. It provides a promising technical foundation for developing high-efficiency, low-cost organic photovoltaics, showing extensive prospects in flexible integration and biomedical applications. To fully capitalize on these prospects, the performance and stability of NIL-patterned flexible OPVs must be rigorously compared and benchmarked against the best non-patterned flexible counterparts, ensuring that the added complexity of nanopatterning translates into a net benefit in real-world application metrics [80].

### Organic Field-Effect Transistors

**Fig. 9:**
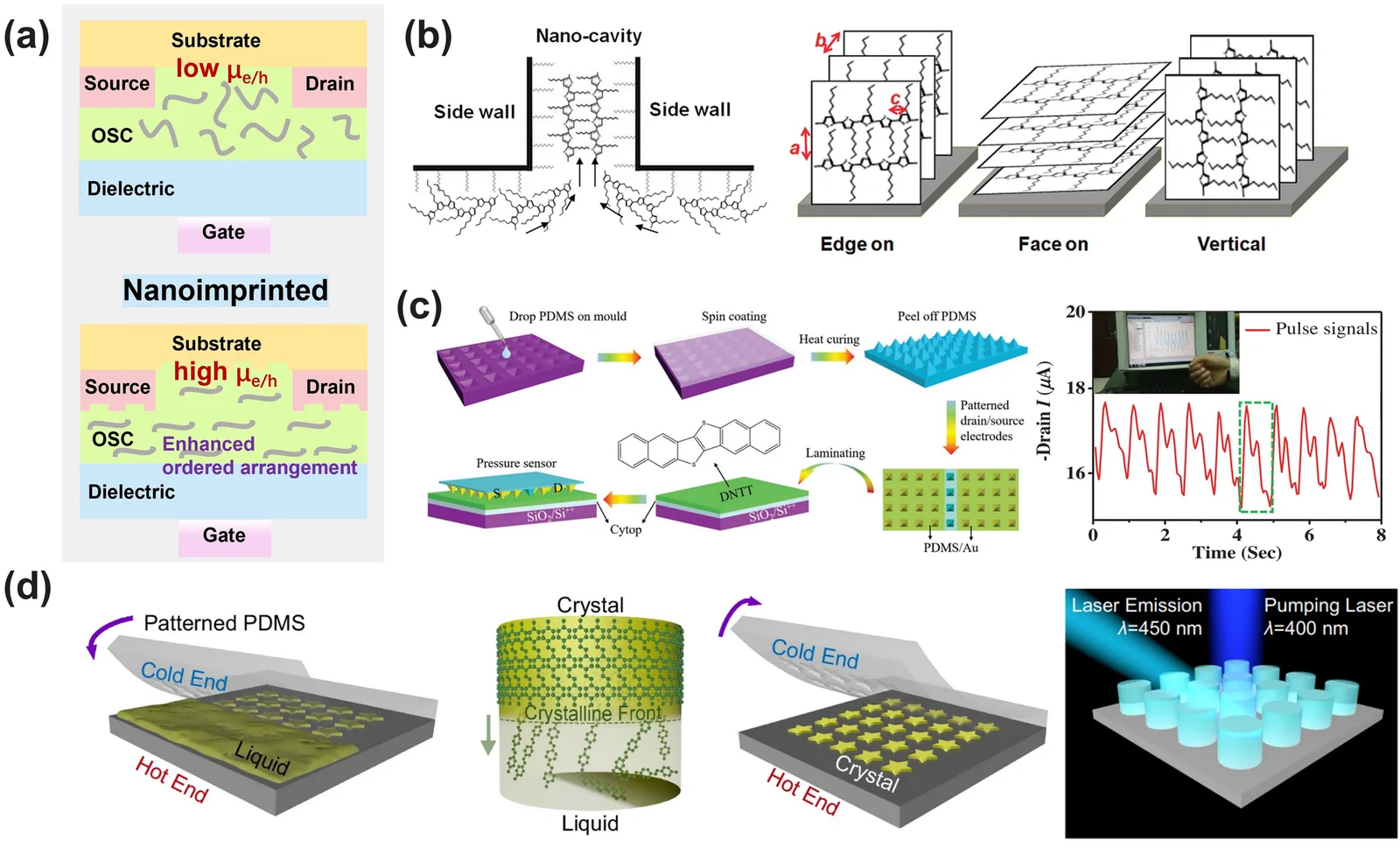
Application of nanoimprinting in OFETs. **a** Schematic of the performance enhancement mechanism in an OFET achieved via NIL. **b** Schematic of P3HT orientation within the mold cavity during imprinting, showing lateral, face-in, and vertical alignment of surface P3HT chains. Reproduced with permission. [62] Copyright 2009, American Chemical Society. **c** A microstructured PDMS with patterned Au designed as a drain/source, then laminated onto the semiconductor layer to form an OFET-based sensor device. The right panel shows the electrical signal induced by the radial artery pulse and its amplified image. Reproduced with permission. [55] Copyright 2019, Wiley. **d** Organic powder is loaded onto the target substrate as raw material. An electric heating wire controls the substrate temperature. After the liquid–solid phase transition and demolding, crystalline organic solar cell microstructures are obtained. Reproduced with permission. [56] Copyright 2025, Springer Nature

OFETs show broad application prospects due to their high sensitivity, reusability, good portability, low power consumption, and room-temperature, low-cost production [161]. However, issues such as relatively low charge carrier mobility, slow operating speed, and insufficient current density limit their further development[162]. NIL has emerged as a promising technical pathway to help address these challenges. Through precise control of the chain orientation and crystal structure of semi-crystalline polymers, combined with optimization of temperature, pressure, and spatial confinement conditions, NIL offers the potential to enhance device performance. A schematic diagram illustrating the performance enhancement mechanism for NIL-fabricated OFETs is shown in Fig. 9a, with specific performance comparison data summarized in Table 4.

**Table 4:** Performance Enhancement of OFETs via NIL Technology [55, 56, 62, 163–172]

Imprinted Layer	Period	Height	Enhancement	References
Au film	10 μm	-	produces a high sensitivity up to 514 kPa^−1^	[55]
OMSB	–	436 nm-15 μm	1.5 times enhancement of carrier mobility, exhibit good lasing performance and low device-to-device variation (as low as 2%)	[56]
P3HT	200 nm	200–250 nm	*π* − *π* stacking along the nanograting can enhance charge-carrier mobility for FETs, while the vertical chain alignment in nanopillars and nanogratings can enhance vertical charge transport in solar cells	[62]
P3HT	100 nm	75 nm	-	[163]
PVDF-TRFE	400 nm	95 nm	a strongly decreased coercive field, and accelerated ferroelectric switching	[164]
P(VDF-TrFE)	140 nm	30 nm	the coercive field decreased by a factor of 5 compared with that of polycrystalline thin films and bulk samples	[165]
Au film	-	280 nm	all solution-processed OFETs with micrometer to submicrometer critical feature resolution were fabricated in a fully maskless sequence	[166]
graphene	–	–	excellent performance with a high mobility of 0.36 cm^2^ V^−1^ s^−1^and on/off ratio of 10^4^ was measured	[167]
rGO	–	–	maintaining the good device performance and the resulting capability of forming electrodes for complementary circuits through a single procedure	[168]
LOR/NIL resist	–	–	OFETs are fabricated with device cutoff frequencies f > 1 MHz at low operating bias V_DS_ = − 15 V	[169]
SAM	–	2.14 nm	*p*-type dinaphtho[2,3-b:2′,3′-f]thieno[3,2-b]thiophene (DNTT) and n-type F_16_CuPc OFETs show competitive mobility as high as 3 and 0.018 cm^2^ V^−1^ s^−1^, respectively	[170]
PMMS	–	–	the typical *p*-type characteristic with a mobility of 1 × 10^–2^ cm^2^ V^−1^ s^−1^	[171]
SP-1	5 μm	125 nm	the average mobility is 1.11 cm^2^ V^−1^ s^−1^ with a maximum up to 1.64 cm^2^ V^−1^ s^−1^	[172]

#### Molecular Structure Control

A foundational application of NIL in OFETs is the direct manipulation of molecular ordering and nanostructure within the semiconductor layer, which governs charge transport. Aryal et al. [62] imprinted the conjugated polymer poly(3-hexylthiophene) (P3HT) using NIL technology to construct highly dense, ordered nanostructures, achieving precise control over three-dimensional chain orientation (Fig. 9b). Research indicates that performing partially confined nanoimprinting at temperatures above the glass transition temperature and the crystallization temperature, but below the melting temperature, effectively enhances chain disentanglement during polymer flow into the mold cavities. After this process, the imprinted nanogratings with specific line spacing successfully formed the π-π stacking structure crucial for enhancing OFET charge carrier mobility. This work established that thermal NIL can be used to induce beneficial molecular orientations. A primary challenge is extending this precise thermal control to a broader range of high-mobility organic semiconductors that may have complex thermal transitions.

Subsequently, Ding et al. [163] demonstrated that large-area P3HT nanopillar arrays could be fabricated using a cost-effective and straightforward nanoimprint method under solvent-swollen plasticized conditions at room temperature. Furthermore, the solvent-assisted room-temperature nanoimprint technique enabled the formation of face-on-oriented P3HT nanopillars, where the π-π stacking structure of P3HT is aligned perpendicular to the substrate—a configuration particularly advantageous for organic photovoltaic applications. The mold’s nanohole diameter determined this alignment. This solvent-assisted approach highlights a milder route to achieve molecular alignment, mitigating thermal damage. However, the presence of residual solvent and its control during imprinting can affect film uniformity and final device performance.

These results underscore NIL as a versatile tool for tailoring both the nanostructure and molecular orientation of polymeric materials, offering new insights into structure–property relationships. Collectively, they exemplify the strategy of using NIL’s mechanical confinement to dictate molecular packing, a strategy whose effectiveness depends on meticulous optimization of process parameters for each specific material system.

#### Ferroelectric Property Optimization

Beyond charge transport, NIL also enables control over the nanostructure and properties of ferroelectric polymers for non-volatile memory and sensing elements. Kassa et al. [164] found that NIL can effectively control key parameters such as crystal structure, chain orientation, and grain size, helping to mitigate the decline in polarization switching capability that occurs when ferroelectric polymer films are scaled down. Studies confirm that a simple NIL process can fabricate high-density ferroelectric polymer nanostructure arrays with an integration density exceeding 33 Gbits inch⁻^2^. Across the entire patterned area, the polarization axis (dipole rotation axis) achieved synchronized in-plane alignment, and internal defects in the nanostructures were significantly reduced. The optimized crystal orientation and quality resulted in highly uniform switching characteristics across all units. Each nanounit exhibited a narrow, nearly ideal square hysteresis loop with low energy loss and a coercive field of approximately 10 mV m⁻^1^, representing performance metrics that are superior to those reported for unpatterned bulk materials. This illustrates how NIL’s confinement can be used to engineer ferroelectric domains. A remaining challenge is integrating these high-quality nanostructures with other active components in functional circuits.

#### Sensors and Multifunctional Integration Systems

In sensor applications, OFET-based pressure sensors are promising for electronic skin due to their signal amplification. NIL is key to fabricating the essential microstructures. Wang et al. [55] demonstrated the application of the piezoresistive effect at the semiconductor/conductor interface to a pressure sensor, activating this effect through gate modulation (Fig. 9c). They used a silicon mold to create elastic PDMS with micropyramid structures and deposited gold electrodes via shadow masking, forming a coupled electrode structure. The microstructured PDMS with patterned gold electrodes was designed as the drain/source and then laminated onto a semiconductor layer to form a complete OFET sensor device. This sensor cleverly combines the transistor’s piezoresistive effect and gate modulation effect, demonstrating a high sensitivity of 514 kPa⁻^1^, a load response time of 1.8 ms, an unload response time of 6.7 ms, and excellent durability exceeding 10,000 measurement cycles. In practical applications, the sensor successfully detected wrist movement and captured weak pulse signals. Its sensor array could accurately distinguish pressure distributions over large areas, showcasing the great potential of NIL technology for manufacturing large-scale biomimetic electronic skin. This work exemplifies a strategy that uses NIL to fabricate key micromechanical structures for pressure transduction. Challenges for advancing such sensors include achieving uniform response across very large-area arrays and ensuring the long-term robustness of the soft imprinted structures under cyclic loading.

Li et al. [56] aimed to address issues such as high defect density, poor uniformity, and significant device-to-device variation by optimizing a residue-free NIL process. They employed an innovative method involving heating powder to a liquid phase and rationally managing the temperature gradient during molding, achieving in situ control of crystallization kinetics. This enabled the growth of high-quality OSC micro/nanopatterns with low defect density and high uniformity over large-area substrates (Fig. 9d). These patterned molecular crystals with precise geometric control and high crystallinity provide an ideal platform for microlasers. OFET devices fabricated based on this technique showed a 1.5-fold increase in charge carrier mobility and a device-to-device variation of only 2.12%, demonstrating excellent performance consistency. This represents a distinct strategy of using NIL for direct crystal growth and patterning. A key challenge lies in extending this precise crystallization control to a broader range of organic semiconductor materials beyond the model system studied.

These research findings collectively show that NIL technology can create complex, residue-free patterns with high uniformity over large areas, contributing to the development of next-generation organic electronic and photonic systems. The cited studies achieve notable performance within their specific contexts. To gauge NIL’s overall competitiveness for sensor and integrated system manufacturing, its benefits—such as concurrent microstructure definition and material property control—should be quantitatively evaluated against other patterning methods in terms of final device performance, production throughput, and cost [54]. Future advancements will depend on resolving material-process compatibility issues, improving replication fidelity, and, crucially, devising scalable schemes for integrating NIL-patterned components into complete, functional systems.

### Disparity in NIL Development across Organic Optoelectronics

While NIL has demonstrated transformative potential for OLEDs, OPVs, and OFETs, the trajectory and maturity of its development across these domains are markedly asymmetric. This uneven landscape is not indicative of a fundamental limitation of NIL but rather arises from a complex interplay of distinct material constraints, device-specific functional imperatives, and divergent paths to commercialization [54, 102]. A key differentiator lies in the thermal and chemical compatibility between the NIL process and the active materials. OLEDs and OPVs often employ delicate organic small molecules or blends with low glass transition or decomposition temperatures, making thermal degradation a significant concern. Consequently, low-temperature processes like UV-NIL are often the preferred route for these devices. In contrast, the polymeric semiconductors (e.g., P3HT) and dielectric layers common in OFETs generally exhibit greater thermal stability, better accommodating the thermal cycles intrinsic to Thermal NIL, which is leveraged for its superior crystallinity control [62, 151]. The pursuit of even higher performance has introduced new complexities, as seen in emerging perovskite-organic tandem cells, where the stringent thermal and chemical constraints of the perovskite layer pose a formidable challenge for direct NIL patterning. This underscores a broader trend: the rise of organic–inorganic hybrid materials (e.g., perovskites, sol–gel nanocomposites). While their superior optoelectronic properties are highly desirable, their successful integration with NIL demands even more refined process windows, often favoring ultra-gentle techniques like room-temperature electrochemical NIL or carefully modulated sol–gel imprinting [107, 173, 174]. Thus, the material compatibility landscape for NIL is continuously evolving, pushing the technology to adapt beyond its origins in pure organic systems.

Beyond material compatibility, the core functional objective of each device type dictates vastly different patterning requirements, thereby favoring specific NIL approaches. For OLEDs, the paramount goal is light outcoupling, which drives the need for large-area, periodic, or quasi-random nanostructures, such as gratings or microlenses [104]. Here, optical precision is critical, while demands on electrical continuity across the patterned layer are relatively relaxed. OPVs, aiming for maximal light trapping and absorption, benefit from structures like nanogratings and nanopillars, where pattern fidelity and high aspect ratios are essential for efficient light scattering and waveguide modes [175–177]. Hybrid materials, such as nanostructured perovskites, introduce an additional dimension in which NIL can simultaneously pattern the light-management structure and influence the crystallization of the active material, blurring the line between optical and morphological control. The challenge shifts fundamentally for OFETs, where performance hinges on charge transport optimization at the semiconductor–dielectric interface [62, 178]. This demands not only high resolution for channel definition but also atomic-level interfacial smoothness and precise control over molecular orientation—requirements that push NIL to its limits in terms of defect control and pattern fidelity.

Finally, the scale and direction of industrial investment have profoundly shaped NIL’s development focus in each sector. The burgeoning research field of hybrid perovskite photovoltaics and LEDs, while not yet at the mass-production scale of OLEDs, has created a significant new driver for developing compatible, low-damage NIL processes [105, 108, 179]. Here, the impetus is less about immediate throughput and more about achieving the nanostructuring precision necessary to reach theoretical efficiency limits in lab-scale devices—a development pattern reminiscent of OFETs but with different material constraints. The massive, established market for OLED displays has generated substantial impetus for scalable NIL manufacturing, particularly favoring the high-throughput combination of UV-NIL with R2R processes for producing light-extraction films and metasurfaces [79, 84]. Similarly, the strong market pull for flexible and wearable electronics aligns perfectly with NIL’s capability for patterning on non-planar substrates, accelerating its development for OPV applications [53, 106]. In comparison, while OFET-based sensors and circuits represent a growing and vital niche, the current market volume and immediate industrial throughput requirements are lower. This has resulted in a different innovation ecosystem, where NIL research for OFETs remains largely at the laboratory scale, focused more on demonstrating performance breakthroughs and novel device physics rather than on solving mass-production challenges. Thus, the observed disparity in NIL’s development ultimately reflects its adaptive evolution across three distinct contexts defined by unique material landscapes, device physics, and economic realities.

## Conclusion and Outlook

NIL has established itself not merely as a patterning tool, but as a transformative platform for structural engineering in organic optoelectronics. This review details its journey from a novel replication concept to a technology capable of defining the performance frontier for OLEDs, OPVs, and OFETs. The central thesis that emerges is that NIL’s most significant impact lies in resolving a fundamental tension in the field: the conflict between the need for intricate, nanoscale features to manipulate light and charge, and the imperative for low-cost, large-area, flexible fabrication. By mechanically embossing functional nanostructures directly into device layers, NIL bypasses the scalability limits of conventional lithography and the stochastic nature of self-assembly, offering a unique “top-down precision with bottom-up compatibility” pathway.

To fully gauge NIL’s value proposition and industrial relevance, it must be contextualized within the broader ecosystem of nanopatterning and scalable manufacturing technologies. Compared to advanced photolithography, NIL offers a compelling route to sub-100-nm features over large areas without the need for complex optics and expensive light sources, albeit with different trade-offs in overlay accuracy and direct processing of multilayer stacks. Compared with bottom-up methods such as directed self-assembly or colloidal templating, NIL provides deterministic control over arbitrary (including aperiodic) patterns, which are crucial for specific light-management designs, though it requires a prefabricated master. When benchmarked against high-throughput printing techniques (e.g., inkjet, gravure), NIL delivers superior resolution and feature fidelity but may face challenges in rapid design iteration and material deposition versatility. Therefore, NIL is not a universal solution but a strategically vital technology for applications that demand its combined strengths—nanoscale resolution, deterministic patterning, material-agnostic compatibility, and proven scalability via R2R. This niche is prominently occupied by next-generation flexible and organic optoelectronic devices, justifying its role as a “core enabler” while acknowledging the complementary value of other techniques in the manufacturing toolkit.

The core advantage of NIL is its multifaceted ability to intervene at the critical nexus of structure, property, and function in organic devices. This manifests as: (i) Precision light management through deterministic photonic crystals, gratings, and metasurfaces to trap light in OPVs or extract it from OLEDs; (ii) In situ control of material order by templating crystallization and molecular orientation to enhance charge mobility in OFETs and OPVs; and (iii) Facilitation of system integration by enabling the patterning of transparent electrodes, sensor microstructures, and optical components on flexible, non-planar substrates. These capabilities are not device-specific but represent cross-cutting strategic leverages that NIL provides across organic optoelectronics.

However, the relationship between NIL and organic optoelectronics is also defined by inherent tensions and trade-offs that delineate both its current limitations and future research directions. The primary conflict stems from the mismatch between the mechanical and thermal demands of the imprint process and the inherent fragility of organic (and emerging perovskite) materials. This core challenge cascades into specific hurdles across the development cycle: achieving high-fidelity replication without damaging functional layers, ensuring uniformity over meter-scale areas, maintaining mold integrity for thousands of cycles, and integrating NIL steps into complete device fabrication flows without compromising yield or throughput.

The trajectory from laboratory breakthrough to industrial mainstay will be determined by addressing challenges in a sequence that mirrors technology readiness levels:

1. Material-Process Co-Design: The highest priority is developing next-generation organic and hybrid materials with enhanced thermal and mechanical stability, tailored explicitly for NIL processing. Concurrently, NIL variants (e.g., low-pressure UV and electrochemical) must be refined to be gentler. This co-evolution is essential to resolve the fundamental mismatch between process demands and material fragility.
2. Scalability and Process Control: The focus must shift from demonstrating feasibility on centimeter-scale samples to solving engineering problems in true R2R or large-plate production. This necessitates establishing rigorous control over key parameters: achieving and maintaining defect densities acceptable for the target application (e.g., much lower for displays than for some photovoltaic textures), significantly extending mold lifetimes to meet cost-of-ownership targets, and implementing in-line metrology for real-time process monitoring and yield management.
3. Integration and Standardization: NIL must evolve from a standalone step to a seamlessly integrated module within standardized manufacturing lines for OLEDs or OPVs. This requires industry-wide collaboration to establish process protocols, equipment interfaces, and comprehensive cost-of-ownership models that fairly benchmark NIL against incumbent patterning techniques. The success of integration will hinge on throughput matching, contamination control, and overall system reliability across the entire production flow.

The future of outlook for NIL is therefore bifurcated into two parallel, equally critical pathways. The first is continued academic innovation, exploring its role in patterning emerging materials (e.g., 2D semiconductors, metal halide perovskites), creating novel 3D device architectures, and enabling quantum photonic structures. The second, and arguably more decisive, is industrial translation, which demands rigorous benchmarking against key metrics: production speed, defect density, overall equipment effectiveness, and final cost per patterned area. Success in this arena will not come from NIL alone, but from its evolution within a holistic manufacturing ecosystem for flexible electronics.

In conclusion, NIL stands at a pivotal point. It has conclusively proven its scientific merit as a powerful enabler of high-performance organic optoelectronic devices. The coming decade will determine its commercial legacy. By strategically confronting the specific challenges of material compatibility, scalable fidelity, and economic integration, the organic electronics community can transition NIL from a versatile laboratory tool into a cornerstone manufacturing technology, thereby accelerating the arrival of a new generation of intelligent, flexible, and ubiquitous optoelectronic systems.
